# A Novel Ecological Approach Reveals Early Executive Function Impairments in Huntington’s Disease

**DOI:** 10.3389/fpsyg.2019.00585

**Published:** 2019-03-22

**Authors:** Filipa Júlio, Maria J. Ribeiro, Miguel Patrício, Alexandre Malhão, Fábio Pedrosa, Hélio Gonçalves, Marco Simões, Marieke van Asselen, Mário R. Simões, Miguel Castelo-Branco, Cristina Januário

**Affiliations:** ^1^Faculty of Psychology and Education Sciences, University of Coimbra, Coimbra, Portugal; ^2^Coimbra Institute for Clinical and Biomedical Research, Faculty of Medicine, University of Coimbra, Coimbra, Portugal; ^3^Coimbra Institute for Biomedical Imaging and Translational Research, University of Coimbra, Coimbra, Portugal; ^4^Center for Research in Neuropsychology and Cognitive Behavioral Intervention, Faculty of Psychology and Education Sciences, University of Coimbra, Coimbra, Portugal; ^5^Institute of Nuclear Sciences Applied to Health, University of Coimbra, Coimbra, Portugal; ^6^Faculty of Medicine, University of Coimbra, Coimbra, Portugal; ^7^Coimbra University Hospital, Coimbra, Portugal

**Keywords:** Huntington’s disease, executive functions, activities of daily living, ecological validity, virtual reality

## Abstract

**Introduction:** Impairments in executive functions are common in neurogenetic disorders such as Huntington’s disease (HD) and are thought to significantly influence the patient’s functional status. Reliable tools with higher ecological validity that can assess and predict the impact of executive dysfunction in daily-life performance are needed. This study aimed to develop and validate a novel non-immersive virtual reality task (“EcoKitchen”) created with the purpose of capturing cognitive and functional changes shown by HD carriers without clinical manifestations of the disease (Premanifest HD), in a more realistic setting.

**Materials and Methods:** We designed a virtual reality task with three blocks of increasing executive load. The performance of three groups (Controls, CTRL; Premanifest HD individuals, HP; Early Manifest HD patients, HD) was compared in four main components of the study protocol: the EcoKitchen; a subjective (self-report) measure – “The Adults and Older Adults Functional Assessment Inventory (IAFAI)”; the “Behavioural Assessment of Dysexecutive Syndrome battery (BADS)”; and a conventional neuropsychological test battery. We also examined statistical associations between EcoKitchen and the other executive, functional and clinical measures used.

**Results:** The HD group showed deficits in all the assessment methods used. In contrast, the HP group was only found to be impaired in the EcoKitchen task, particularly in the most cognitively demanding blocks, where they showed a higher number of errors compared to the CTRL group. Statistically significant correlations were identified between the EcoKitchen, measures of the other assessment tools, and HD clinical features.

**Discussion:** The EcoKitchen task, developed as an ecological executive function assessment tool, was found to be sensitive to early deficits in this domain. Critically, in premanifest HD individuals, it identifies dysfunction prior to symptom onset. Further it adds a potential tool for diagnosis and management of the patients’ real-life problems.

## Introduction

Huntington’s disease (HD) is a neurodegenerative genetic movement disorder mainly characterized by subcortical pathology involving the basal ganglia and the frontostriatal circuitry, with prominent cell loss and atrophy in the caudate and putamen (Shoulson and Young, 2011). Testing positive for HD only indicates that someone carries the gene defect, does not equate to having the disease (O’Keeffe et al., 2009), as the test result does not inform about how and when the symptoms will start, nor about the current disease status (Dumas et al., 2013). Individuals who carry the genetic mutation but who do not yet meet the criteria for an HD clinical diagnosis are considered to be in a premanifest HD phase. The conversion from a premanifest to a manifest HD stage is traditionally based on the onset of unequivocal motor symptoms. Nevertheless, cognitive, behavioral and neuroanatomical changes have been reported to occur before any clinically detectible motor signs (Aylward et al., 2004; Rosas et al., 2005; Rosenblatt, 2007; Paulsen, 2010; Roos, 2010).

Huntington’s disease clinical presentation includes motor, behavioral and cognitive alterations that typically arise in middle adulthood, when family and career responsibilities are often greatest (Nehl et al., 2004). Impairments in executive functions are frequent in HD affected individuals, even in premanifest or early manifest disease stages (Novak and Tabrizi, 2010; Paulsen, 2011; Stout et al., 2011; Tabrizi et al., 2011) and are thought to significantly influence their functional status and to be major contributors to everyday deficits, disability and loss of autonomy (Godefroy et al., 2010; Stout et al., 2011; Reilmann et al., 2014; Ross et al., 2014a). The executive dysfunction associated with HD includes deficits in planning and multitasking, sequencing, set-shifting, attentional control, response inhibition and perseveration (Rosenblatt, 2007; Novak and Tabrizi, 2011; Dumas et al., 2013). These changes are thought to reflect HD brain sequelae, namely the disruption of the frontal-subcortical, and specifically, prefrontal-striatal circuitry, and the altered functioning of brain circuits that are important for organizing behavior, cognitive flexibility, the planning of an instrumental performance, response inhibition, attention, and temporal control over motor output (Rosas et al., 2005; Balci et al., 2009; Paulsen, 2010; Rao et al., 2014). These executive deficits need to be properly acknowledged and assessed as they can have a considerable impact on the quality of life and daily functioning of HD affected individuals (Hamilton et al., 2003; Nehl et al., 2004; Hoth et al., 2007; Beglinger et al., 2010; Mörkl et al., 2016).

Executive functions can be defined as the “capacities that enable a person to engage successfully in independent, purposive, self-serving behavior” (Lezak et al., 2012). These complex, higher-order abilities are needed to be able to adapt in a flexible manner to many daily life situations that require task conceptualization, planning, action and evaluation (Dumas et al., 2013). As executive functioning requires so many integrated cognitive functions and supervisory processes, impairments in this domain tend to be supramodal and affect the expression of all aspects of behavior (Lezak, 1982). To drive a car, pay bills, take the medication at the right time of the day, prepare a meal – these are all examples of Instrumental Activities of Daily Living (IADL) that involve executive functions, and these are exactly the kind of activities that are reportedly impaired early in the course of HD (Beglinger et al., 2010; Williams et al., 2011), even when individuals show a relatively unimpaired performance in conventional executive tests or present average scores in functional measures such as the widely used Total Functional Capacity scale (TFC) (Shoulson and Fahn, 1979). As Lezak (1982) states, impairments in executive functions can compromise a person’s capacity to maintain an independent and productive life no matter how well he can see and hear, walk, and talk, and perform tests. This seems to apply perfectly to the premanifest HD condition, where changes in day-to-day function are more likely to be experienced in tasks that require multiple cognitive, motor, and behavioral abilities (Williams et al., 2011), such as doing routine work, manage finances or drive safely (Beglinger et al., 2010), rather than in the performance of single and more abstract tests.

In fact, the subtle changes in behavior and cognition observed in individuals who do not yet display disease-related motor alterations (premanifest HD stage) and early manifest HD individuals are often missed in highly structured examinations (Stout et al., 2007, 2016; Reilmann et al., 2014), as traditional cognitive and functional measures seem insensitive to the initial changes in HD. Backing this idea, a thorough review of studies about cognition in HD by Dumas et al. (2013) found almost equal support for and against the presence of executive deficits in premanifest gene carriers, highlighting the need for further research. Moreover, in neuropsychology, few objective methods for assessing the functional impact of executive impairments are available, as traditional tests measure cognitive abilities in isolated and artificial situations, which bear little similarity to the situations that patients encounter in their daily life (Chaytor et al., 2006; Allain et al., 2014). These type of clinical tools are urgently needed to demonstrate daily life functional changes besides cognitive efficacy as evaluated by classical neuropsychological testing (Royall et al., 2007), so that the success of interventions can be progressively evaluated in terms of the effects they have on quality of life and functional independence, and not merely in terms of efficacy in reducing primary symptoms (Moore et al., 2007). Therefore, new, more ecological, and more sensitive assessment tools that are able to document the insidious onset of subtle executive alterations in the daily functioning of HD affected individuals and that are able to demonstrate changes in day-to-day function in HD, and specifically in premanifest HD, are urged (Downing et al., 2014).

To address these issues and understand the inconsistencies often found between the results obtained in formal examinations and the real-life complaints about the cognitive and functional status of premanifest and early manifest HD individuals, a new assessment tool was created at our Lab: EcoKitchen, a non-immersive virtual reality task consisting of preparing meals in a kitchen. EcoKitchen was based in two main premises: on the one hand, cooking is a good example of a real-world task that often draws heavily on executive functioning (Tanguay et al., 2014); on the other hand, different assessment and rehabilitation studies of clinical populations have successfully used kitchen settings to address functional and executive impairments (Baum and Edwards, 1993; Zhang et al., 2003; Craik and Bialystok, 2006; Bialystok et al., 2008; Giovannetti et al., 2008; Allain et al., 2014; Ruse et al., 2014). Notably, the EcoKitchen improves on the existing tools for several reasons: it is more portable and standardized than some of the methods that are done in real kitchens or involve manipulating props (e.g., Baum and Edwards, 1993; Giovannetti et al., 2008); outputs combine time and error measures, whereas some existing methods rely more heavily on only one dimension and omit valuable information about the changes in speed/accuracy trade-off often seen in clinical populations (e.g., Craik and Bialystok, 2006; Giovannetti et al., 2008); focus more on the examinee and less on the examiner, having less observational bias and less external cues that can prompt action or improve action correctness (Zhang et al., 2003; Ruse et al., 2014); it informs about the impact of increasing executive load on the participants behavior, having different levels of complexity (e.g., Allain et al., 2014); has higher realism, as the virtual scenario created tried to include known food and beverage brands and more life-like stimuli than previous studies (e.g., Craik and Bialystok, 2006).

The inclusion of real-world scenarios and virtual reality tasks in clinical studies might provide a good mean to evidence the impact of executive impairment on the patients’ life (Albani et al., 2010; Frisch et al., 2012) – delivering sensitive measures of everyday function and a valid testing ground to assess the impact of executive deficits in daily-life (Moore et al., 2007; Poliakoff and Smith-Spark, 2008; Frisch et al., 2012). Few studies have used or developed performance-based tools to assess everyday functioning in HD. Nicoll et al. (2014) used the “Memory for Intentions Screening Test” as a standardized performance-based measure of prospective memory in HD and Sheppard et al. (2017) used the “Advanced Finances Test” as a performance-based measure of the participants’ ability to manage finances. Both studies were done in semi-naturalistic settings (real materials and props handled in a laboratory) and resorted to observational methods to infer about the mild-moderate HD patients’ performance level. In our view, the EcoKitchen has the potential to increase the objectivity and sensitivity of HD executive and functional assessments such as the ones mentioned for several reasons: it proposes a more refined definition of the executive sub-domains being evaluated; makes a clearer link to conventional executive and functional tools often used in HD clinical practice; evaluates the impact of different executive loads over individual performance as it manipulates the task executive demands; it provides quantitative data about the performance time and accuracy of the examinee.

EcoKitchen was designed to evaluate planning, multi-tasking, set-shifting, cognitive flexibility, self-monitoring, sequencing, divided attention, and scanning skills. As Dumas et al. (2013) stated, it is often very difficult to pinpoint just one specific executive function that is responsible for the correct performance of a task. Moreover, as Craik and Bialystok (2006) indicate, the choice of a real-life task means that the specificity of measurement of individual cognitive functions is limited, as there are no unitary constructs involved in daily-life performance – we think that the same principle might apply to computer-simulated tasks. Consequently, each one of the parameters considered in the EcoKitchen performance analysis can be associated not with one executive function but rather with a sub-set of executive domains. This association is further detailed in the “Materials and Methods” section.

To our knowledge, this is the first study where a virtual reality task was created specifically for the assessment of functional deficits related to executive impairments in HD patients, particularly in premanifest HD individuals. Thus, this work was essentially planned as an exploratory feasibility study, aimed at checking if EcoKitchen was well-tolerated by the clinical groups and if it was able to differentiate healthy participants from HD affected individuals, particularly premanifest HD participants. Furthermore, this study aimed to identify which variables computed from the EcoKitchen task might be more sensitive to the earliest disease-related cognitive and functional alterations and, thus, withhold the potential to be used in clinical, research and rehabilitation settings in the future.

We tested the executive function of HD, HP and healthy Controls using the newly developed EcoKitchen tool and compared the results with other executive and functional measures in a wider assessment protocol that included a subjective (self-report) functional measure – “The Adults and Older Adults Functional Assessment Inventory (IAFAI)” (Sousa et al., 2015), a more ecological executive test battery – the “Behavioural Assessment of Dysexecutive Syndrome (BADS)” (Wilson et al., 1996), and a conventional neuropsychological test battery. The “IAFAI” (Sousa et al., 2015) was used as a subjective (self-report) verbal functional measure, since it was considered to be more accurate and broad than other subjective functional assessments, namely the Total Functional Capacity scale. The “BADS” (Wilson et al., 1996) was included in the protocol as a more close to daily-life like situations traditional executive tool (Wilson et al., 1998; Norris and Tate, 2000). Importantly, to our knowledge, BADS has been scarcely used with HD patients (Nimmagadda et al., 2011), with no reports in premanifest HD found, and this is the first time IAFAI was used with HD affected individuals.

Finally, the conventional neuropsychological battery, composed by a set of widely known executive tests, was used as a description of the executive status of the premanifest and early manifest HD participants enrolled in this study. The tests included in this battery were chosen because they were proven to be sensitive to basal ganglia damage and HD earliest cognitive changes (Lemiere et al., 2002; Paulsen, 2010; Lezak et al., 2012; Dumas et al., 2013). Moreover, these tests are extensively used in clinical settings (some are part of the cognitive section of the main standardized HD assessment scale, the Unified Huntington’s Disease Rating Scale – Huntington Study Group, 1996) and in large multinational longitudinal observational studies [e.g., Registry Study (Baake et al., 2017); TRACK-HD (Tabrizi et al., 2009); PREDICT-HD (Paulsen et al., 2006); HD-CAB (Stout et al., 2014)]. Finally, the executive sub-domains assessed by these tests are thought to correspond to the executive sub-domains involved in EcoKitchen task performance and, thus, we expected performance in both components of our study protocol to be correlated, helping us to describe the cognitive skills elicited by the EcoKitchen.

In summary, this study aimed to test EcoKitchen as a new performance-based tool to detect the earliest signs of executive and functional changes in HD prior to the onset of clinical symptoms and overcome the limitations often posed by the traditional assessment methods.

## Materials and Methods

### Participants

A total of 15 Early Manifest Hutington’s disease participants (HD), 15 Premanifest Huntington’s disease participants (HP), and 19 Control participants (CTRL) completed the four protocol components and entered the data analysis, after the exclusion of one HD and one CTRL participants for presenting a score in the “Montreal Cognitive Assessment (MoCA)” (Nasreddine et al., 2005; Freitas et al., 2011) below the established cut-off for their age and education level. Due to time constraints, two CTRL participants did not complete IAFAI.

HD and HP participants were recruited from the Movement Disorders Unit of the Neurological Department of Coimbra University Hospital. All but three CTRL participants were gene negative or non-at-risk relatives of the HD affected participants. All subjects gave written informed consent in accordance with the Declaration of Helsinki to participate in the study approved by our Institutional Ethics Committees (Faculty of Medicine and Coimbra University Hospital).

The participants were assigned to one of three groups according to the following criteria:

(1) Early Manifest HD (HD): patients with mild HD symptoms – stages I–II (Shoulson and Fahn, 1979), that had a UHDRS Total Functional Capacity scale of 10–13 and a positive HD genetic test result which confirms a CAG length of ≥36 (*n* = 15).
(2) Premanifest HD (HP): participants that showed no clinical symptoms of HD, that had a UHDRS Total Motor score ≤5 and a positive HD genetic test result which confirms a CAG length of ≥36 (*n* = 15).
  Exclusion criteria for the clinical groups included dementia, severe depression, history of substance abuse, and any other neurological condition.
(3) Controls (CTRL): healthy participants, with no history of dementia, depression, substance abuse, any neurological and/or psychiatric condition and no current use of psychotropic medication (*n* = 19).

The clinical groups were assessed by an experienced neurologist using the “Unified Huntington’s Disease Rating Scale” (UHDRS) – Motor and Total Functional Capacity scales (Huntington Study Group, 1996). The UHDRS Total Motor score can range from 0 to 124 and higher scores indicate increased severity of motor symptoms. The UHDRS Total Functional Capacity scale can range from 0 to 13 – lower scores indicate increased disability. Disease duration was defined for each early manifest HD participant as the number of years since HD clinical diagnosis. Langbehn’s formula (Langbehn et al., 2004) was used to calculate the estimated time (in number of years) to disease onset of the HP participants, although no further classification of the premanifest participants was done according to this parameter. Information about the CAG repeat number was collected for both clinical groups.

To avoid cognitive confounds, we used MoCA (Nasreddine et al., 2005; Freitas et al., 2011) as a mild cognitive impairment and dementia screening tool and excluded any subjects that were below the expected score on this test. The “Beck Depression Inventory-II” (Beck et al., 1996; Campos and Gonçalves, 2011) was used as a neuropsychiatric measure and also as an exclusion criterion if moderate to severe depressive symptoms were signaled. The “Irregular Word Reading Test (TeLPI)” (Alves et al., 2012) was administered to provide an estimate of the level of premorbid intelligence of all the participants. The “Edinburgh Handedness Inventory” (Oldfield, 1971) was used to define subject’s handedness.

The demographic characteristics of the three groups are presented in Table 1.

**Table 1:** Demographic characteristics with Kruskal–Wallis and Mann–Whitney comparisons across groups.

	CTRL = 19	HP = 15	HD = 15	K–W	M–W	M–W	M–W
	Gender	Gender	Gender		HP vs. CTRL	HD vs. CTRL	HP vs. HD
	(F:M) 15:4	(F:M) 12:3	(F:M) 10:5
	Handedness	Handedness	Handedness
	(R:L) 18:1	(R:L) 15:0	(R:L) 14:1				
	
Demographic	Median	Median	Median	χ^2^	*U*	*U*	*U*
characteristics	(IQR; min–max)	(IQR; min–max)	(IQR; min–max)	(*p*-value)	(*p*-value)	(*p*-value)	(*p*-value)
Age (years)	41	36	46	6.075^∗^	95	112.5	56 #
	(12; 25–57)	(16; 22–52)	(6; 25–69)	(0.048)	(0.099)	(0.297)	(0.019)
Education (years)	11	14	9	6.582^∗^	111	95	53.5 #
	(7; 6–17)	(7; 6–17)	(6; 6–16)	(0.037)	(0.270)	(0.095)	(0.013)
CAG	–	42	43	–	–	–	97.5
		(5; 39–49)	(2; 38–50)				(0.529)
Disease duration (years)	–	–	5	–	–	–	–
			(6; 1–10)				
Years to HD onset	–	16.46	–	–	–	–	–
		(10.43; 7.37–43.34)					
UHDRS – TFC	–	13	12	–	–	–	45 #
		(0; 13–13)	(2; 10–13)				(0.001)
UHDRS – Motor	–	0	24	–	–	–	1 #
		(3; 0–5)	(20; 5–44)				(<0.001)
MoCA	26	27	23	10.218^∗^	119	67 ¥	45 #
	(4; 22–30)	(4; 21–30)	(4; 18–29)	(0.006)	(0.411)	(0.008)	(0.005)
BDI-II	3	6	17	8.715^∗^	121.5	62 ¥	61 #
	(4; 0–24)	(9; 0–23)	(17; 0–23)	(0.013)	(0.464)	(0.005)	(0.032)
TeLPI (QIEC)	113.54	116.60	103.18	8.303^∗^	116.5	78.5 ¥	49 #
	(15.35; 67.44–125.82)	(13.05; 91.64–126.09)	(19.98; 84.72–121.98)	(0.016)	(0.367)	(0.026)	(0.008)
TeLPI (QIV)	114.83	117.88	103.28	8.592^∗^	117.5	79.5 ¥	46 #
	(15.89; 71.76–127.67)	(14.31; 94.31–127.67)	(19.68; 87.01–123.88)	(0.014)	(0.385)	(0.029)	(0.006)
TeLPI (QIR)	109.72	111.72	102.51	8.230^∗^	118.5	78.5 ¥	49 #
	(11.25; 70.58–118.91)	(9.19; 91.16–118.91)	(16.45; 84.98–115.82)	(0.016)	(0.405)	(0.026)	(0.008)


*CTRL, control; HP, premanifest HD; HD, early manifest HD; IQR, interquartile range; min–max, minimum and maximum scores (range); K–W, Kruskal–Wallis; M–W, Mann–Whitney; CAG, CAG repeat expansion confirmed by a genetic test; Disease duration, years since HD clinical diagnosis (only computed for HD); years to HD onset, years to estimated HD clinical diagnosis according to Langbehn’s formula (only computed for HP); UHDRS – TFC, Total Functional Capacity scale of the Unified Huntington’s Disease Rating Scale; UHDRS – Motor, Motor scale of the Unified Huntington’s Disease Rating Scale; MoCA, Montreal Cognitive Assessment; BDI-II, Beck Depression Inventory II; TeLPI, The Irregular Word Reading Test; QIEC, Full Scale Intelligence Quotient; QIV, Verbal Intelligence Quotient; QIR, Performance Intelligence Quotient. ^∗^Significant group effect (Kruskal–Wallis, p < 0.05). ^¥^HD≠Control (Mann–Whitney, p < 0.05); ^#^HP≠HD (Mann–Whitney, p < 0.05).*

The study protocol included four different components: EcoKitchen, IAFAI, BADS, and a conventional neuropsychological test battery.

### EcoKitchen

As previously mentioned, EcoKitchen is a new assessment tool created at our laboratory in order to add performance-based information to the other executive and functional measures used. EcoKitchen is a non-immersive virtual reality task that aims to objectively evaluate the cognitive and functional status of the study participants using a realistic scenario – a computer-generated kitchen.

#### EcoKitchen Design and Procedures

EcoKitchen was implemented on a desktop PC, with 23″ monitor (large screen size of 23-inch), in full screen mode (1920 × 1200). The stimuli were generated with Vizard (WorldViz) Virtual Reality – version 4.0. The participant experienced the kitchen environment from a first-hand perspective and used the computer mouse to move around the scenario.

EcoKitchen was designed as a non-immersive desktop computer task, which involves a flat-screen presentation of the virtual kitchen setting. This option in comparison with a fully immersive virtual reality display has several advantages. It is more portable than a three-dimensional environment, thus facilitating assessment in clinical settings (Allain et al., 2014); it limits the risk of simulation sickness, which could pose a problem for elderly participants or clinical groups (Attree et al., 1996; Kawano et al., 2012); it is more appropriate for individuals less familiarized with computers; finally, it creates very little memory demands to the study participants – individuals did not need to navigate through the scenario, having the risk of forgetting where the requested items were.

**FIGURE 1:**
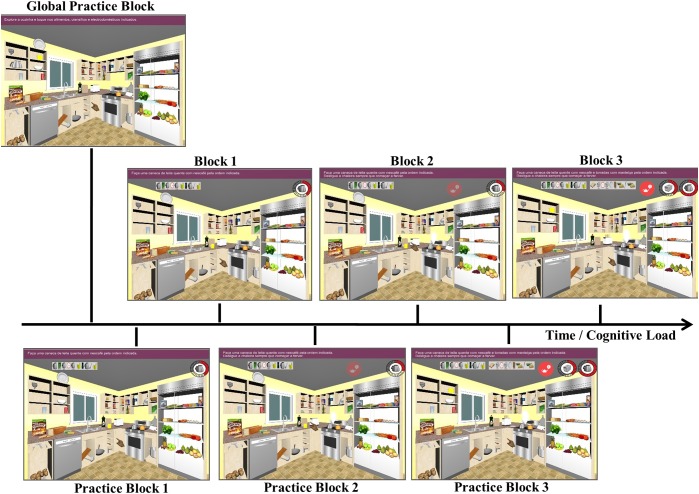
EcoKitchen task design. EcoKitchen included three different blocks (each preceded by a practice trial), with increasing executive demands. In Block 1, the participant had to prepare a cup of coffee with milk – Task A. In Block 2, while performing Task A, the participant had to turn off a boiling kettle that burst at several random moments. In Block 3, the participant had to perform the tasks previously described, whilst preparing toasts with butter (Task B).

The task included three different blocks, with an increasing executive load. Each condition was preceded by a practice block. There was also a first global practice block, to guarantee that each participant was completely familiarized with the apparatus before the assessment blocks begun – see Figure 1.

Global Practice Block – The participant had to explore the kitchen environment and grab a specific list of items – all the items that he/she was going to need in the following blocks were on that list, as well as distracter items.
Block 1 – A picture list with all the items needed to prepare a cup of coffee with milk (Task A) was displayed on the upper part of the screen. The list was left in full view during the block to reduce constraints on memory. Participants were instructed to collect each item, in the order they appeared in the list, as fast and accurately as possible. They had to attend and turn off the stove as soon as and only when the clock was completely red. Participants had to plan and monitor their behavior to complete this level successfully.
Block 2 – Participants had to perform Task A, as described in the first block, while, simultaneously, paying attention and monitoring a boiling kettle that was on the stove. They were instructed to press the kettle every time and as soon as smoke came out and a red signal appeared on the right upper part of the screen, to prevent water from spilling. The kettle was set up to burst three times during the block, at random moments, so participants had to check smoke and the red signal appearance periodically. Participants had to recruit the same executive skills as before, plus divide their attention to complete this level successfully.
Block 3 – Participants had to perform the tasks described in the first and the second blocks (Task A and boiling kettle). Additionally, participants were instructed to also prepare toasts with butter (Task B). A picture list with all the items needed to prepare the second snack was displayed on the upper part of the screen. Participants were instructed to alternate between the two lists (Task A and Task B) to make sure that both tasks were completed at the same time. Participants had to apply the same skills used in the previous block, plus switch/alternate between tasks to complete this level successfully.

The interactions with the different items needed to perform the tasks were facilitated – e.g., if the participant touched the jar, it would go automatically to the stove. This was settled not only to help the participants who globally did not have much experience with computer interaction, but also to focus on the cognitive aspects of task performance rather than on the motor coordination/control aspects – which can be problematic in a movement disorder. EcoKitchen aimed to analyze executive functioning while minimizing the impact of computer interaction difficulties (Allain et al., 2014). To reduce memory constraints, the instructions and the lists with the requested items and actions needed to perform either Task A or Task B were left in full view during the whole block. With the same purpose, there were no closed cabinets or drawers in EcoKitchen, all items were on full display. Finally, to increase the realism of the task (and thus its ecological validity), known commercial brands were used to depict the foods and beverages included in the kitchen setting.

#### EcoKitchen Data Analysis

Several parameters were defined for the analysis of the EcoKitchen performance of each participant, considering Time and Error variables. Although the performance of tasks that simulate daily-life routines requires a plethora of cognitive functions and executive sub-domains that are difficult to disentangle, we have added some information about the executive functions that, in our view, are reflected by each EcoKitchen parameter.

• Performance Time Task A – The time the participant was engaged in the preparation of a cup of coffee with milk (time elapsed from the moment the first item of the list was picked to the moment the last item of the list was picked and used). This parameter reflects psychomotor and processing speed, planning, and motor time.
• Performance Time Task B – The time the participant was engaged in the preparation of toasts with butter (time elapsed from the moment the first item of the list was picked to the moment the last item of the list was picked and used). This parameter reflects the same executive domains as Performance Time Task A, plus task switching.
• Reaction Time Stove – The amount of time the participant took to react and turn off the stove once the clock was completely red (which was the cue for the behavior to take place and for the participant to initiate the response). This parameter gives indications about behavior monitoring, response initiation, divided attention, and set-shifting.
• Reaction Time Kettle – The amount of time the participant took to react and turn off the kettle once smoke appeared and a red signal blinked in the right upper part of the computer screen (which were the cues for the behavior to take place and for the participant to initiate the response). This parameter reflects divided attention, sustained alertness, response initiation, and set-shifting.
• Reaction Time Toaster – The amount of time the participant took to react and turn off the toaster once the clock was completely red (which was the cue for the behavior to take place and for the participant to initiate the response). This parameter gives indications about the same executive domains tackled by Reaction Time Stove, plus task switching.
• Reaction Time per Block – The mean of the different reaction times extracted from each EcoKitchen block. This parameter reflects all the executive sub-domains involved in the different reaction times to specific cues.
• Sequencing Errors – The number of times the participant failed to follow the proper sequence of the task (e.g., tried to mix the coffee with the spoon before adding the milk). This parameter reflects planning, behavior monitoring, and working memory.
• Item Errors – The number of times the participant picked items of the EcoKitchen scenario that were not needed to prepare either Task A or Task B (e.g., selected a pineapple instead of coffee). This parameter reflects attention and behavior monitoring.
• Impulsivity Errors Stove – The number of times the participant tried to turn off the stove before the proper time (before the clock being completely red). This parameter reflects response inhibition or inhibitory control, and attention.
• Impulsivity Errors Toaster – The number of times the participant tried to turn off the toaster before the proper time (before the clock being completely red). This parameter reflects the same executive sub-domains involved in Impulsivity Errors Stove, plus task switching.
• Total Errors/Performance Time Task A – The number of errors per minute the participant did during the completion of Task A (cup of coffee with milk). This parameter gives indication about the speed–accuracy balance in task completion.

$$\frac{\text{total errors}}{\text{performance time task A}\times 60}$$

• Total Errors/Performance Time Task B – The number of errors per minute the participant did during the completion of Task B (toasts with butter). This parameter gives the same indication as the previous one, plus indication about task switching abilities.

$$\frac{\text{total errors}}{\text{performance time task B}\times 60}$$

### IAFAI – The Adults and Older Adults Functional Assessment Inventory

We have used “IAFAI” (Sousa et al., 2015) as a verbal and subjective measure of the functional status of study participants. In IAFAI, the participant must rate his level of self-perceived difficulties in performing Basic and Instrumental Activities of Daily Living (BADL and IADL, respectively), such as bathing, using an ATM card or cooking a meal. Each activity can have a score of 0 (representing the absence of difficulty/dependence in the execution of the ADL) or a score of 1 (representing the presence of difficulty/dependence in the execution of the ADL) (Sousa et al., 2015). Moreover, the participant must indicate if each of the signaled difficulties is explained by physical, cognitive, or emotional restrictions. Seven incapacity percentages were computed from IAFAI: Global Functional Incapacity (GFI), Functional Incapacity in Basic Activities of Daily Living (ABVD), Functional Incapacity in Household Instrumental Activities of Daily Living (H-IADL), Functional Incapacity in Advanced Instrumental Activities of Daily Living (A-IADL), Functional Incapacity due to Physical Factors (Physical), Functional Incapacity due to Cognitive Factors (Cognitive), and Functional Incapacity due to Emotional Factors (Emotional).

### BADS – The Behavioural Assessment of Dysexecutive Syndrome Battery

For the executive functions assessment, we used the “BADS” (Wilson et al., 1996), created by Barbara Wilson to overcome the ecological validity constraints of other traditional executive tests (Burgess et al., 1998, 2006). This battery is composed by six sub-tests, all of which imply skills and materials that try to resemble daily-life like situations. Seven variables were extracted from BADS: Total Score, Rule Shift Cards Test Score, Action Program Test Score, Key Search Test Score, Temporal Judgement Test Score, Zoo Map Test Score, and Modified Six Elements Test Score.

### Neuropsychological Test Battery

The conventional neuropsychological test battery used as a baseline description of the executive status of the study participants assembled several classic executive tests widely employed in clinical and research settings. The Phonemic Verbal Fluency test: three letters – P, M, R (Cavaco et al., 2013a) and the Semantic Verbal Fluency test – category animals (Cavaco et al., 2013a) were used to assess working memory, word generation and inhibition. The Stroop test – Naming, Interference and Reading tasks (Stroop, 1935) were used to assess cognitive flexibility and processing speed. The Symbol Digit Modalities Test (Smith, 1982) was used to assess working memory, attention and integration, and psychomotor speed. The Digit Span Test (Forward and Backward) of the WAIS-III – Wechsler Adult Intelligence Scale-third edition (Wechsler, 1997, 2008) was used to assess working memory. The Trail Making Test – parts A and B (Cavaco et al., 2013b) was used to assess scanning, sequencing, divided attention, psychomotor speed and cognitive flexibility. Finally, the Wisconsin Card Sorting Test (Heaton, 1981) was used to assess abstract behavior and set-shifting. All the tests were applied in a strictly prescribed order, to avoid any interference effects or content overlapping. Twenty scores were extracted from this test battery.

### Statistical Analyses

**FIGURE 2:**
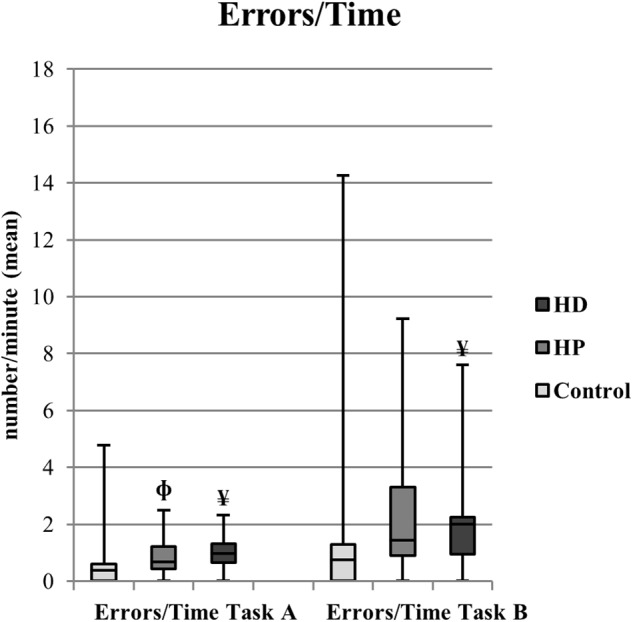
EcoKitchen Number of Total Errors per minute of Performance Time Task A and Number of Total Errors per minute of Performance Time Task B (mean) and significant differences between groups. Boxplots: central mark – median; edges of box – 25th and 75th percentiles; whiskers – most extreme data points (minimum and maximum). ^Φ^HP≠Control (Mann–Whitney, *p* < 0.05); ^¥^HD≠Control (Mann–Whitney, *p* < 0.05).

**FIGURE 3:**
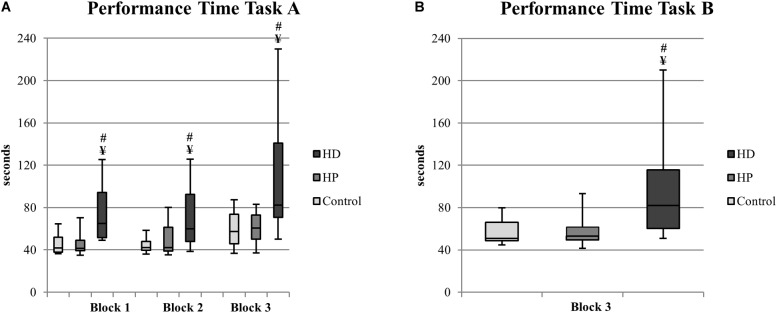
EcoKitchen Performance Time Task A **(A)** and Performance Time Task B **(B)** and significant differences between groups. Boxplots: central mark – median; edges of box – 25th and 75th percentiles; whiskers – most extreme data points (minimum and maximum). ^¥^HD≠Control (Mann–Whitney, *p* < 0.05); ^#^HD ≠ HP (Mann–Whitney, *p* < 0.05).

**FIGURE 4:**
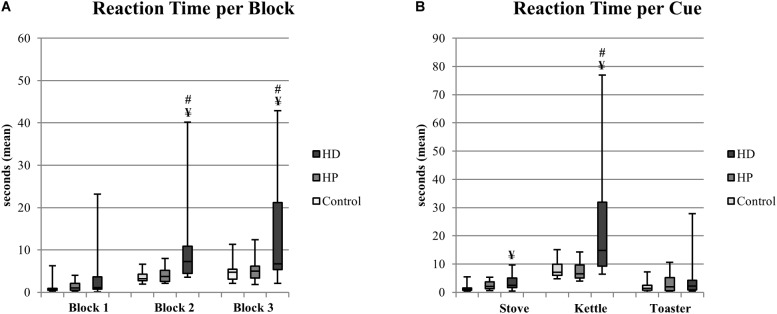
EcoKitchen Reaction Time per Block **(A)** and Reaction Time per Cue **(B)** – Stove, Kettle, and Toaster (mean of the three blocks) and significant differences between groups. Boxplots: central mark – median; edges of box – 25th and 75th percentiles; whiskers – most extreme data points (minimum and maximum). ^¥^HD≠Control (Mann–Whitney, *p* < 0.05); ^#^HD≠HP (Mann–Whitney, *p* < 0.05).

Comparisons of quantitative variables between the three groups (HD, HP, and Controls) were performed resorting to Kruskal–Wallis tests. When statistically significant differences were detected (effect of group), *post hoc* comparisons were performed between two groups using the Mann–Whitney *U* tests. Comparisons of nominal/categorical variables between groups were performed resorting to Chi-square tests of independence. Wilcoxon-Signed rank tests were used to analyze the effects of the increasing cognitive load in the participants’ performance across the three EcoKitchen blocks. Spearman rank correlation coefficients were calculated to examine the associations of EcoKitchen, the other assessment methods and HD features for the clinical groups (HP and HD). Benjamini–Hochberg corrections with false positive rate established at 0.05 were used to deal with multiple comparisons, and only the correlations that survived these corrections were mentioned in the “Results” section and further examined in the “Discussion” section. To reduce the number of pairwise correlations and enhance interpretability, in the correlation analyses, the variables related to EcoKitchen were averaged across the three Blocks. In the correlations with clinical variables, it is of note that disease duration (in years) was only considered for the early manifest HD group (*n* = 15) and estimated years to likely onset was only considered for the premanifest HD group (*n* = 15), thus reducing the sample size considered for computing the correlation coefficients in this case. Finally, given the high intra-group variability detected in the EcoKitchen performance of the early manifest HD participants (as reflected in the boxplots depicted in Figure 2–4), there was a possibility that the differences observed in our study were driven by just a few patients within this group. In order to test this hypothesis, we checked for outliers using the following logical conditions [(*x*_i_ ≥ Q_3_ + 1.5 ^∗^ IQR) and (*x*_i_ ≤ Q_1_ - 1.5 ^∗^ IQR)]. For each of the EcoKitchen variables computed there was a maximum of two outliers within the HD group. In total, six of the 15 early manifest HD patients enrolled in our study presented outlier results in at least one of the EcoKitchen computed measures, but there was no participant whose performance was identified as an outlier in all variables. Moreover, this sub-group of patients was demographically and clinically matched to the other early manifest HD participants (*p* ≥ 0.05 in all the variables displayed in Table 1). Lastly, no HD outliers were found on four of the EcoKitchen measures, namely, in Performance Time Task A (Block 1 and Block 2), in Reaction Time per Block (Block 3), and in Reaction Time per Cue (Stove). Kruskal–Wallis and Mann–Whitney *U* comparisons were performed as described above excluding the outliers identified in the HD group. Importantly, the statistical results were equivalent to the results obtained including all data points. As high intra-group variability reflects the phenotypic variability that is reportedly one of the key features of this disease (Folstein et al., 1984; Waldvogel et al., 2012; Mehrabi et al., 2016), these early manifest HD outliers were considered to be clinically and scientifically relevant, and therefore we decided not to exclude them from our main analyses presented in this paper. All calculations were performed with IBM SPSS Statistics 24, adopting a level of significance of α = 0.05.

## Results

### EcoKitchen

The several time and error variables extracted from the participants’ performance in the EcoKitchen task gave us important indications about how well HD affected individuals could perform executively demanding tasks similar to daily-life routines and, thus, about their functional status. Moreover, the analysis of the EcoKitchen data gave us relevant information about the impact of increasing executive load on the behavior of clinical and healthy populations. We defined three main categories for the EcoKitchen data analysis: accuracy, time, and cognitive load. Only significant results are reported.

#### EcoKitchen Accuracy Measures

##### EcoKitchen errors

We computed three types of errors from the EcoKitchen performance data – sequencing errors (participant failed to follow the proper sequence of the task), item errors (participant picked items not needed to complete the task), and impulsivity errors (participant turned off the stove or toaster before the proper time). Sequencing errors reflected difficulties in the planning and monitoring of actions. We found a significant group effect for the percentage of participants with sequencing errors in Block 1 and Block 3 of EcoKitchen [(χ^2^(2) = 13.253, *p* = 0.001) and (χ^2^(2) = 8.964, *p* = 0.011), respectively] – see Table 2. Interestingly, *post hoc* tests comparing the different groups revealed that a significantly higher percentage of HP participants than controls failed to plan and correctly sequence their actions, but only in the more executive challenging EcoKitchen Block 3 [χ^2^(1) = 4.437, *p* = 0.035]. The early manifest HD group also showed more sequencing errors than controls in Block 1 and Block 3 [χ^2^(1) = 9.188, *p* = 0.002, and χ^2^(1) = 6.689, *p* = 0.010, respectively], and was also worse than HP participants in Block 1 [χ^2^(1) = 8.889, *p* = 0.003]. We also found a significant group effect in terms of impulsivity errors in Block 3 of EcoKitchen [χ^2^(2) = 12.621, *p* = 0.002]. This might reflect deficits in inhibitory control and increased impulsivity in the early manifest HD group, as a higher percentage of HD than CTRL and HP participants tried to stop the stove before the proper time in the more cognitively demanding EcoKitchen condition [(χ^2^(1) = 7.425, *p* = 0.006) and (χ^2^(1) = 6.000, *p* = 0.014), respectively].

**Table 2:** Percentage of participants that had a score ≠ 0 in the Sequencing, Item and Impulsivity Error Variables of the EcoKitchen Task.

	CTRL			HP			HD		
			
EcoKitchen	Block 1	Block 2	Block 3	Block 1	Block 2	Block 3	Block 1	Block 2	Block 3
Sequencing Errors	15.8	26.3	52.6	13.3	20	86.7 Φ	66.7 ¥#	40	93.3 ¥
Item Errors	0	0	5.3	6.7	6.7	20	6.7	20	13.3
Impulsivity Errors – Stove	10.5	5.3	0	6.7	6.7	0	13.3	20	33.3 ¥#


*CTRL, control; HP, premanifest HD; HD, early manifest HD. ^Φ^HP≠Control (Chi-square test for independence – p < 0.05); ^¥^HD≠Control (Chi-square test for independence – p < 0.05); ^#^HD≠HP (Chi-square test for independence – p < 0.05).*

##### EcoKitchen errors/time

We calculated the number of errors per minute as a measure of speed–accuracy trade-off in each group of participants. We found a significant group effect in the number of errors per minute during the preparation of a cup of coffee with milk [χ^2^(2) = 8.174, *p* = 0.017] – see Figure 2. Importantly, in the *post hoc* tests, HP participants showed a decrease in the quality of their task performance, committing more errors per minute during Task A than controls (*U* = 86, *p* = 0.048). The early manifest HD patients also showed a diminished quality of their task performance, as they presented a higher number of sequencing, item and impulsivity errors per minute than controls during both Task A and Task B completion (*U* = 65.5, *p* = 0.007 and *U* = 80.5, *p* = 0.029, respectively).

#### EcoKitchen Time Measures

##### EcoKitchen performance time

We analyzed and compared the time it took the participants from the three groups to prepare a cup of coffee with milk (Task A) and toasts with butter (Task B), to see whether group differences would emerge. We found a statistically significant group effect in Task A and Task B performance times in all EcoKitchen conditions [Task A: Block 1 – χ^2^(2) = 21.972, *p* < 0.001; Block 2 – χ^2^(2) = 12.512, *p* = 0.002; Block 3 – χ^2^(2) = 13.959, *p* = 0.001; Task B: χ^2^(2) = 16.475, *p* < 0.001] – see Figure 3. Notably, we found no differences between HP and control participants in total task time suggesting that the motor and cognitive times of premanifest individuals were not affected. In contrast, we found that early manifest HD patients already displayed a motor and cognitive slowness that influenced their timely performance both in single and multitasking conditions, as they were slower compared to controls [Task A: Block 1 – *U* = 25, *p* < 0.001; Block 2 – *U* = 46, *p* = 0.001; Block 3 – *U* = 46, *p* = 0.001; Task B: *U* = 41, *p* < 0.001], and to premanifest participants [Task A: Block 1 – *U* = 14, *p* < 0.001; Block 2 – *U* = 46, *p* = 0.006; Block 3 – *U* = 38, *p* = 0.002; Task B: *U* = 27, *p* < 0.001] across all EcoKitchen Blocks and Tasks.

##### EcoKitchen reaction time

We analyzed the amount of time participants took to react to the different cues included in the EcoKitchen scenario (stove, kettle, and toaster). These reaction time measures reflect cognitive functions like response initiation, monitoring, divided attention, set-shifting and task switching skills. The average reaction time across the different cues presented a significant group effect in the more demanding EcoKitchen Blocks 2 and 3 [χ^2^(2) = 13.680, *p* = 0.001 and χ^2^(2) = 7.727, *p* = 0.021, respectively] – see Figure 4. No differences were found between the HP and CTRL groups. Reversely, HD patients took longer than CTRL and HP participants to react to the target stimuli while engaged in a primary task, even when prompt action indications were given. We found that the HD group was significantly slower than CTRL and HP participants in Blocks 2 and 3 (*U* = 38.5, *p* < 0.001 and *U* = 68, *p* = 0.010; *U* = 48, *p* = 0.007 and *U* = 60.5, *p* = 0.031, respectively). When considering the EcoKitchen cues separately, again a significant group effect was found in the reaction times to turn off the stove and to turn off the boiling kettle [χ^2^(2) = 9.152, *p* = 0.010 and χ^2^(2) = 14.458, *p* = 0.001, respectively]. HD patients showed a slower response initiation to attend the stove and the kettle than controls (*U* = 59, *p* = 0.004 and *U* = 51, *p* = 0.002, respectively), and were also slower than HP participants to react to the kettle (*U* = 33, *p* = 0.001). Interestingly, there was a trend for the HP group to be slower to turn off the stove than controls (*U* = 86.5, *p* = 0.052), although this did not reach statistical significance.

#### EcoKitchen Cognitive Load

Next, we analyzed the effects of increasing cognitive load in the participants’ performance across the three EcoKitchen blocks to see whether the behavior of study participants reflected the impact of the task executive demands. The incremental complexity of EcoKitchen had a negative impact in performance accuracy, as we found a significant increase in the percentage of participants that failed to follow the proper sequence of the task in Block 3 compared to Blocks 1 and 2 (*Z* = -4.690, *p* < 0.001 and *Z* = -4.271, *p* < 0.001, respectively). This was paralleled by an increase in the number of errors per minute during the completion of Task A in Block 3 compared to Blocks 1 and 2 (*Z* = -5.077, *p* < 0.001 and *Z* = -3.972, *p* < 0.001, respectively). The increasing cognitive demands of EcoKitchen also had a negative impact in terms of timing measures, as the time participants devoted to Task A in Block 3 was significantly higher than in Block 1 and Block 2 (*Z* = -5.287, *p* < 0.001, *Z* = -5.217, *p* < 0.001, respectively). Finally, the reaction time to the stove cue also reflected the EcoKitchen increasing complexity, as it was significantly higher in Block 3 compared to Blocks 1 and 2 (*Z* = -3831, *p* < 0.001 and *Z* = -4.153, *p* < 0.001, respectively).

### IAFAI – The Adults and Older Adults Functional Assessment Inventory

The IAFAI results gave us relevant information about the self-reported functional status of study participants and about the insight the clinical groups have about their ability to perform different daily-life tasks. We found that a higher percentage of early manifest HD patients than CTRL and HP participants report difficulties in the performance of both BADL and IADL – see Table 3. Namely, a significantly higher percentage of HD than CTRL and HP participants signaled functional difficulties in basic activities of daily living, in household IADL, and in advanced IADL. Furthermore, a significantly higher percentage of HD than CTRL and HP participants attributed the cause of the experienced functional difficulties to physical, cognitive, and emotional factors. These results suggest that early manifest HD patients have insight about the deficits they experience when performing simple and complex daily-life tasks, as well as about the factors that might be causing these deficits. Importantly, HP and CTRL individuals rated themselves equally capable, with no statistically significant differences between the two groups on any of the variables extracted from IAFAI. Thus, HP participants are not aware of functional changes in their daily routines, perceiving their everyday performance at the control level and reporting significantly less difficulties than HD participants in all of the IAFAI measures considered.

**Table 3:** Percentage of participants that had a score ≠ 0 in IAFAI (The Adults and Older Adults Functional Assessment Inventory).

				Chi-square	Chi-square	Chi-square
	CTRL	HP	HD	HP vs. CTRL	HD vs. CTRL	HP vs. HD
	
IAFAI	%			χ^2^	χ^2^	χ^2^
				(p-value)	(p-value)	(p-value)
Global Functional	17.6	26.7	93.3	0.379	18.331 ¥	13.889 #
Incapacity				(0.538)	(<0.001)	(<0.001)
Functional Incapacity	0	20	80	3.752	21.760 ¥	10.800 #
in Basic ADL				(0.053)	(<0.001)	(0.001)
Functional Incapacity	0	13.3	93.3	2.418	28.207 ¥	19.286 #
in Household IADL				(0.120)	(<0.001)	(<0.001)
Functional Incapacity	17.6	13.3	80	0.112	12.441 ¥	13.393 #
in Advanced IADL				(0.737)	(<0.001)	(<0.001)
Functional Incapacity –	0	20	80	3.752	21.760 ¥	10.800 #
Physical Factors				(0.053)	(<0.001)	(0.001)
Functional Incapacity –	11.8	13.3	93.3	0.018	21.208 ¥	19.286 #
Cognitive Factors				(0.893)	(<0.001)	(<0.001)
Functional Incapacity –	11.8	13.3	60	0.018	8.219 ¥	7.033 #
Emotional Factors				(0.893)	(0.004)	(0.008)


*CTRL, control; HP, premanifest HD; HD, early manifest HD; IAFAI, the Adults and Older Adults Functional Assessment Inventory; BADL, Basic Activities of Daily Living; IADL, Instrumental Activities of Daily Living. ^¥^HD≠Control (Chi-square test for independence – p < 0.05); ^#^HD≠HP (Chi-square test for independence – p < 0.05).*

### BADS – The Behavioural Assessment of Dysexecutive Syndrome Battery

The BADS results were important to determine if a neuropsychological test battery with higher ecological validity than conventional executive tests could be better at differentiating the clinical and control groups. We found a statistically significant group effect in the Total Score and in several of the subtests that comprise it, namely in the Rule Shift Cards Test, the Action Program Test, and the Zoo Map Test – see Table 4. Early manifest HD patients presented lower scores when compared to controls and premanifest participants in all these subtests. Notably, HP and CTRL participants presented similar scores in all the computed BADS measures, which suggests that even with tasks that try to simulate daily-life executive demands, the HP group did not differ from CTRL participants.

**Table 4:** BADS (the Behavioural Assessment of Dysexecutive Syndrome battery) results with Kruskal–Wallis and Mann–Whitney comparisons across groups.

					M–W	M–W	M–W
	CTRL	HP	HD	K–W	HP vs. CTRL	HD vs. CTRL	HP vs. HD
	
	Median	Median	Median	χ^2^	*U*	*U*	*U*
BADS	(IQR; min–max)	(IQR; min–max)	(IQR; min–max)	(*p*-value)	(*p*-value)	(*p*-value)	(*p*-value)
Total	17	18	12	16.985^∗^	130	31 ¥	36 #
	(3; 13–22)	(6; 12–21)	(5; 9–18)	(<0.001)	(0.662)	(<0.001)	(0.001)
Rule Shift Cards Test	4	4	3	9.676^∗^	140	76 ¥	50 #
	(1; 1–4)	(1; 3–4)	(2; 0–4)	(0.008)	(0.916)	(0.013)	(0.005)
Action Program Test	4	4	3	11.069^∗^	129	69 ¥	66 #
	(0; 2–4)	(0; 0–4)	(2; 1–4)	(0.004)	(0.447)	(0.002)	(0.029)
Key Search Test	3	2	2	5.110	97.5	82.5 ¥	102.5
	(2; 0–4)	(2; 0–4)	(2; 0–4)	(0.078)	(0.106)	(0.032)	(0.669)
Temporal Judgement Test	2	1	1	3.284	101	103.5	105
	(1; 0–3)	(1; 0–3)	(1; 0–2)	(0.194)	(0.123)	(0.137)	(0.731)
Zoo Map Test	2	3	1	10.632^∗^	103	74 ¥	44 #
	(2; 0–4)	(2; 0–4)	(2; 0–3)	(0.005)	(0.159)	(0.014)	(0.004)
Modified Six Elements Test	4	4	3	4.656	139.5	91.5	72.5
	(1; 1–4)	(1; 2–4)	(2; 0–4)	(0.097)	(0.906)	(0.056)	(0.073)


*CTRL, control; HP, premanifest HD; HD, early manifest HD; IQR, interquartile range; min–max, minimum and maximum scores (range); BADS, The Behavioural Assessment of Dysexecutive Syndrome battery; K–W, Kruskal–Wallis; M–W, Mann–Whitney. ^∗^Significant group effect (Kruskal–Wallis, p < *0.05). ^¥^HD≠Control (Mann–Whitney, p* < *0.05); ^#^HD≠HP (Mann–Whitney, p* < 0.05)*

### Neuropsychological Test Battery

The results obtained in the conventional neuropsychological tests applied gave us a comprehensive picture about the participants’ cognitive status in the different executive sub-domains tapped by this battery. We observed a statistically significant group effect in several of the executive measures applied, namely in the Phonemic Verbal Fluency – PMR total correct, letter P and letter R correct scores; the Stroop Word Reading, Color Naming and Interference tests; the Semantic Verbal Fluency – total correct; the Symbol Digit Modalities Test – total correct; the Digit Span Test – backward and total scores; the Trail Making Test A and B time measures; and the Wisconsin Card Sorting Test – percentage of errors – see Table 5. The *post hoc* analyses showed us that the early manifest HD patients presented deficits in most of the tests applied when compared to control participants, namely in the Phonemic Verbal Fluency – PMR total correct, the Stroop Word Reading, Color Naming and Interference tests, the Semantic Verbal Fluency – total correct, the Symbol Digit Modalities Test – total correct, the Digit Span Test – backward and total scores, the Trail Making Test A and B time measures, and the Wisconsin Card Sorting Test – percentage of errors and perseverative errors. Statistically significant differences were also found between the HD and HP conventional executive test profiles, namely in the Phonemic Verbal Fluency – PMR total, letter P and letter R; the Stroop Word Reading, Color Naming and Interference tests; the Semantic Verbal Fluency – total correct; the Symbol Digit Modalities Test – total correct; the Digit Span Test – forward, backward and total scores, and the Trail Making Test A and B time measures.

**Table 5:** Neuropsychological Test Battery results with Kruskal–Wallis and Mann–Whitney comparisons across groups.

					M–W	M–W	M–W
	CTRL	HP	HD	K–W	HP vs. CTRL	HD vs. CTRL	HP vs. HD
	
	Median	Median	Median	χ^2^	*U*	*U*	*U*
Neuropsychological Test Battery	(IQR; min–max)	(IQR; min–max)	(IQR; min–max)	(*p*-value)	(*p*-value)	(*p*-value)	(*p*-value)
Phonemic Verbal Fluency – PMR correct	29	35	23	7.634^∗^	115	84.5 ¥	50 #
	(21; 13–59)	(17; 18–59)	(20; 11–46)	(0.022)	(0.340)	(0.044)	(0.009)
Phonemic Verbal Fluency – P correct	12	13	8	7.673^∗^	109	92.5	47 #
	(9; 5–24)	(6; 6–23)	(7; 1–18)	(0.022)	(0.243)	(0.082)	(0.006)
Phonemic Verbal Fluency – M correct	9	12	7	4.383	129	92.5	68
	(8; 4–19)	(4; 5–19)	(8; 2–15)	(0.112)	(0.639)	(0.082)	(0.064)
Phonemic Verbal Fluency – R correct	10	10	7	6.274^∗^	127.5	90.5	53 #
	(6; 3–20)	(5; 6–19)	(6; 2–14)	(0.043)	(0.601)	(0.070)	(0.013)
Stroop Word Reading – correct	88	90	58	20.768^∗^	128.5	22 ¥	24 #
	(23; 53–118)	(21; 50–110)	(17; 20–88)	(<0.001)	(0.627)	(<0.001)	(<0.001)
Stroop Color Naming – correct	68	71	45	21.693^∗^	142	19 ¥	21.5 #
	(15; 40–100)	(23; 45–90)	(10; 26–67)	(<0.001)	(0.986)	(<0.001)	(<0.001)
Stroop Interference – correct	43	48	26	26.001^∗^	90	19 ¥	9.5 #
	(8; 26–62)	(7; 25–60)	(9; 10–37)	(<0.001)	(0.068)	(<0.001)	(<0.001)
Semantic Verbal Fluency – correct	22	20	15	19.954^∗^	108.5	23 ¥	32 #
	(9; 12–36)	(5; 13–39)	(5; 8–19)	(<0.001)	(0.237)	(<0.001)	(0.001)
Symbol Digit Modalities Test – correct	52	56	30	27.584^∗^	122	6.5 ¥	7.5 #
	(12; 35–75)	(19; 30–70)	(13; 16–42)	(<0.001)	(0.476)	(<0.001)	(<0.001)
Symbol Digit Modalities Test – errors	0	0	0	1.540	115	119	112
	(0; 0–5)	(1; 0–2)	(1; 0–4)	(0.463)	(0.248)	(0.310)	(0.981)
Digit Span – forward	8	9	7	4.409	109	110	64 #
	(5; 4–12)	(5; 5–14)	(2; 4–11)	(0.110)	(0.241)	(0.254)	(0.042)
Digit Span – backward	5	6	4	12.176^∗^	138.5	46 ¥	51 #
	(2; 3–10)	(3; 3–11)	(2; 2–7)	(0.002)	(0.887)	(0.001)	(0.010)
Digit Span – total	13	15	11	8.329^∗^	121.5	76 ¥	50 #
	(8; 7–21)	(7; 8–22)	(3; 7–17)	(0.016)	(0.464)	(0.020)	(0.009)
Trail Making Test – part A time	26	24	42	19.098^∗^	129.5	22 ¥	32 #
	(7; 14–41)	(15; 15–63)	(31; 27–74)	(<0.001)	(0.651)	(<0.001)	(0.001)
Trail Making Test – part A errors	0	0	0	1.875	138.5	118.5	96.5
	(0; 0–1)	(0; 0–1)	(1; 0–2)	(0.392)	(0.804)	(0.209)	(0.340)
Trail making test – part B time	62	58	156	21.389^∗^	129.5	18 ¥	24 #
	(33; 36–120)	(31; 36–222)	(97; 64–366)	(<0.001)	(0.652)	(<0.001)	(<0.001)
Trail Making Test – part B errors	0	1	1	3.498	111.5	96	96
	(1; 0–3)	(1; 0–3)	(2; 0–3)	(0.174)	(0.223)	(0.074)	(0.469)
WCST – trials administered	87	86	94	3.785	134.5	87.5	79.5
	(24; 70–128)	(55; 70–128)	(37; 73–128)	(0.151)	(0.780)	(0.056)	(0.169)
WCST – percentage of errors	17.24	16.85	26.61	6.037^∗^	121.5	69.5 ¥	79
	(10.16; 8.57–41.41)	(24.64; 10–52.34)	(14.78; 10.96–41–41)	(0.049)	(0.466)	(0.011)	(0.164)
WCST – perseverative errors	9	11	19	5.592	112	74 ¥	84.5
	(10; 5–29)	(21; 5–51)	(21; 5–42)	(0.061)	(0.288)	(0.017)	(0.244)


*CTRL, control; HP, premanifest HD; HD, early manifest HD; IQR, interquartile range; min–max, minimum and maximum scores (range); K–W, Kruskal–Wallis; M–W, Mann–Whitney; WCST, Wisconsin Card Sorting Test. ^∗^Significant group effect (Kruskal–Wallis, p < 0.05). ^¥^HD≠Control (Mann–Whitney, p < 0.05); ^#^HD≠HP (Mann–Whitney, p < 0.05).*

Interestingly, HP individuals presented a similar performance to controls in all the variables extracted from this battery, showing the same executive profile as healthy participants. The results of the early manifest HD group suggest that tests with time constraints and that assess processing/psychomotor speed are more sensitive to HD executive changes. Furthermore, these results, together with BADS results, seem to indicate that the overall executive status of the HP and CTRL groups is similar, and that any subtle cognitive changes due to HD might remain undetectable with the use of these type of conventional assessment approaches (e.g., paper and pencil tests).

### Correlational Analyses

We have found that the time parameters of EcoKitchen were significantly correlated with the different measures included in the study protocol. Specifically, the time the clinical groups took to prepare the cup of coffee with milk (Performance Time Task A) and to prepare the toasts with butter (Performance Time Task B) and the time they took to react to the different EcoKitchen cues, particularly the boiling kettle (Reaction Time per Block and Reaction Time Kettle), were significantly correlated with their self-reported functional difficulties as measured by IAFAI, except for the Functional Incapacity due to Emotional Factors (all significant correlations *r*_s_ ≥ 0.46, *p* < 0.05). Thus, the subjective perception of the clinical groups about their ability to perform basic and complex activities of daily living is associated with the time features of their objective performance in a simulated kitchen task. The EcoKitchen performance and reaction time parameters were also significantly correlated with several conventional neuropsychological tests, particularly those that had time constraints, such as the Phonemic and Semantic Verbal Fluency Tests, the Stroop Word Reading, Color Naming and Interference Tests, the Symbol Digit Modalities Test – total correct and the Trail Making Test A and B Time measures (all significant correlations |*r*_s_|≥ 0.51, *p* < 0.05). The significant correlations found between the EcoKitchen time measures and the classical neuropsychological tests shed further light on the different cognitive sub-domains involved in EcoKitchen task completion, namely cognitive flexibility, divided attention, sequencing, psychomotor/processing speed, inhibition, and working memory. Finally, the performance time and reaction time of the HP and HD groups in the EcoKitchen were significantly correlated with the Motor and Functional Capacity scores obtained in the UHDRS – Unified Huntington’s Disease Rating Scale (all significant correlations |*r*_s_|≥ 0.52, *p* < 0.05), which suggests that EcoKitchen time measures capture some of the main HD clinical features, namely the motor symptoms severity, and the subjective functional status ascribed by an experienced neurologist. In contrast, no statistically significant correlations were found between the EcoKitchen accuracy parameters and the other executive and functional measures included in the study protocol, nor HD clinical features. Furthermore, no statistically significant correlations were found between any of the EcoKitchen variables and the different BADS sub-scores.

The significant associations found between EcoKitchen and the different well-established assessment methods indicate which functional areas and which executive sub-domains are captured by the time variables computed from this novel virtual tool, adding further validation and clarification about what is being measured during the performance of simulated household tasks. Moreover, the significant correlations found between EcoKitchen and HD symptom severity give important indications about the functional implications of HD clinical phenotype to daily-life like tasks.

## Discussion

The EcoKitchen, a novel virtual reality task that evaluates executive skills and their functional outcome using a performance-based setup, was able to distinguish between HD, HP and control participants. Notably, this novel task showed that HP individuals present diminished accuracy during the performance of daily-life like routines. This study also confirmed that early manifest HD individuals already present cognitive and functional alterations, revealed by all the assessment tools used – EcoKitchen, IAFAI, BADS, and conventional neuropsychological tests.

The difference found between the HP and CTRL participants enrolled in this study is notable because these were high functioning (TFC median = 13) and far from estimated disease onset (Years to Onset median = 16) individuals, and measures that detect functional changes in premanifest HD populations with this clinical profile have been challenging to find (Downing et al., 2014). Nevertheless, these measures are critical to use if relevant lessons are to be drawn from research studies and clinical trials. The identification and quantification of subtle disease-related alterations in individuals that carry the abnormal gene but who do not yet meet the criteria for an HD clinical diagnosis (premanifest HD) provides a window of opportunity for interventions aimed at preventing or delaying symptom onset (Weir et al., 2011). Furthermore, reliable and accurate assessment methods that can discriminate between disease stages and record changes in the persons’ performance are extremely important to clinical, research and rehabilitation settings (Baum and Edwards, 1993).

As noted by Stout et al. (2016), researchers still know very little about how people with HD perform in everyday life, as there have been no studies that examined cognitive performance in the natural setting. While relatively common in other clinical models, like Traumatic Brain Injury or Alzheimer’s Disease (Baum and Edwards, 1993; Zhang et al., 2003; Giovannetti et al., 2008; Allain et al., 2014; Tanguay et al., 2014), few studies have used performance-based tasks to assess function in HD. EcoKitchen was designed to assess the executive skills (like planning, sequencing, scanning, dividing attention, set-shifting or multi-tasking) involved in a routine action such as meal preparation in a controlled performance-based format. Virtual reality technology is considered pivotal to improve the knowledge about the cognitive features underlying functional disability in HD, given the constraints of conventional methods to detect subtle alterations in premanifest HD populations.

In fact, there is an ongoing debate about the low sensitivity and diminished ecological validity of the more traditional neuropsychological tests (Chaytor et al., 2006; Allain et al., 2014; Parsons et al., 2015). Measures of executive function aim to assess a number of constructs: selective attention, inhibitory control, planning, impulsivity, problem solving, and some aspects of short-term memory (Parsons et al., 2015). However, these hypothetical constructs may have little relevance to real-world behaviors (Burgess et al., 2006; Parsons et al., 2015), which can lead to inconsistencies as low scores on classical measures of executive function do not necessarily imply poor executive behavior in everyday life and, conversely, a good performance on classical executive measures can be accompanied by severely dysexecutive behavior in everyday life (Tanguay et al., 2014). While the early manifest HD patients were impaired in all the conventional executive measures used, the HP individuals enrolled in our study presented a similar performance to controls, showing the same executive profile as healthy participants. These results are in line with other studies that did not find significant cognitive differences between far from estimated onset premanifest HD individuals and controls (van Asselen et al., 2012; Dumas et al., 2013; Tabrizi et al., 2013; Baake et al., 2017). Moreover, no differences were found between the HP and CTRL groups in any of the BADS measures, which supports the claim that, although having higher ecological validity than classic executive tests and mimicking real-world situations (Burgess et al., 2006), BADS may not be sensitive to executive impairments in relatively high functioning individuals (Sohlberg and Mateer, 2001). HP and CTRL groups gave similar self-reports of functional status, as assessed by IAFAI. This is in line with the description of Reilmann et al. (2014) of a HD phase where signs and symptoms have only minor impact on the function and where, although some intra-individual decline may occur from the premorbid level of functioning, this is not usually detectable on subjective measures like TFC (or IAFAI, we add). In our study, IAFAI was unable to differentiate a group of premanifest HD participants that already display performance deficits in the EcoKitchen from healthy individuals. Furthermore, the impairments revealed by the IAFAI results of the early manifest HD group suggest that despite lack of insight being often reported in HD (Ho et al., 2006; Sitek et al., 2014), the patients enrolled in our study appeared to be at least self-aware of their functional deficits. This observation is in line with previous findings that showed that in HD, and particularly in earlier stages of HD, self-awareness of functional dysfunction (impairments in the performance of activities of daily living) is better preserved than the self-awareness of the motor, cognitive or psychiatric changes associated with this clinical condition (Snowden et al., 1998; Sitek et al., 2011; McCusker and Loy, 2014).

Robust differences were found between the premanifest individuals and controls in our study in the number of errors in the more cognitively demanding block of EcoKitchen, where multi-tasking, divided attention and set-shifting were required. Also, the fact that the HP group exhibited mainly sequencing errors, reveals the existence of early impairments in planning, behavior monitoring and/or working memory prior to HD clinical diagnosis. The HP participants presented a performance time similar to healthy controls, but this was done at a cost of having an increased number of errors and a trend to show slower reaction times – which might be a reflection of the speed–accuracy trade-off that often occurs in clinical populations (Heitz, 2014). Furthermore, the EcoKitchen performance of HP participants consubstantiates the claim of Williams et al. (2015) that functional changes during the prodromal HD period may well reflect subtle changes in everyday cognitive functioning that predate a motor diagnosis of HD – deficits were observed not in the performance time by itself (which could be linked to early changes in motor speed, for example) rather in the time to react to parallel cues, in the number of errors, and in the number of errors per minute committed, particularly in the EcoKitchen blocks with higher executive load. Thus, the cognitive and functional profile shown by the HP individuals in our study corroborates the allegation of Stout et al. (2016) that the study of functional cognition (how well an individual operates cognitively in everyday life) may allow to observe the effects of HD with greater sensitivity and ecological validity than conventional cognitive and/or functional assessment methods, and that computerized assessments open new horizons for investigating that topic. Similarly to what Allain et al. (2014) suggested for Alzheimer’s disease patients, EcoKitchen demonstrated that virtual reality environments (ecologically valid, controlled and safe scenarios) are a promising alternative that can be used for the detection of everyday action impairments in premanifest and early manifest HD stages. Also, as Keefe et al. (2016) indicate, performance-based functional capacity measures do not involve subjective judgments about one’s own abilities nor require informants, so they might help to reduce the burden on investigators and participants in future clinical studies. Moreover, the IAFAI and EcoKitchen results of the HP group seem to be in line with the findings of Nicoll et al. (2014) in a study about prospective memory in HD, where a discrepancy between the participants’ performance-based and self-reported function was identified. This further highlights the importance of using objective measures to assess the functional and cognitive status of premanifest HD individuals, as the self-report methods to assess function are susceptible to bias and the classic cognitive tests often lack the sensitivity to subtle executive impairments and do not inform about their relevance to everyday performance.

Importantly, the several statistically significant correlations found between EcoKitchen and the other cognitive and functional assessment measures used, as well as HD clinical features, further validate the scope and aim of this novel virtual reality task that we have created for HD affected individuals. Namely, the time variables extracted from EcoKitchen (performance time and reaction time) were significantly correlated with the other protocol components used, except for BADS, as well as with HD clinical features. These statistically significant associations showed us which of the cognitive sub-domains assessed by conventional neuropsychological tests were related to the variables computed from EcoKitchen and, thus, underlie the performance of daily-life like tasks. Our correlational analysis suggests that cognitive flexibility, speed of thinking and acting, divided and sustained attention, scanning, sequencing, and integration skills, inhibitory control and working memory are closely associated with the timely performance of household chores, specifically kitchen tasks. In our view, unraveling the cognitive architecture that frames the performance of routine tasks is extremely relevant for the planning of tailored interventions that aim to prevent, delay or rehabilitate specific functional deficits, as it allows to identify and stimulate the different cognitive sub-domains underlying the function loss. This is in line with the claim of Parsons et al. (2015) that virtual environments may add to an existing neuropsychological battery when attempting to make accurate predictions about a person’s behavior in the real world, as they allow to measure the functional output of constructs within the complexity of a real-world environment. Furthermore, the robust correlations found between IAFAI and EcoKitchen variables signaled the existence of a high convergent validity between the two measures (both seem to assess the same construct – function) and indicate which self-reported daily-life functional incapacities assessed by IAFAI were related to EcoKitchen’s performance (both BADL and IADL were associated with the virtual performance of kitchen chores). As Moore et al. (2007) state, performance-based instruments may be improved by being co-normed with subjective reports of functioning, and this study seems to support this view, as it combines subjective and objective information about the functional skills of study participants. The correlations found between the EcoKitchen parameters and HD clinical variables, such as the severity of motor symptoms and the patients’ functional capacity rated by the neurologist (UHDRS – Motor and TFC scores) gave us indications about which disease features were most strongly associated with the behavior of the HD and HP groups on this novel functional task, and corroborate the thoroughly reported increasing functional incapacity due to motor impairments associated with this condition (Rothlind et al., 1993; Ross et al., 2014b). Finally, the lack of correlation between the error variables computed from EcoKitchen and the other assessment methods that integrated the study protocol suggests that the EcoKitchen provides additional information regarding the cognitive and functional status of HD affected individuals that is not conveyed by the other methods.

In sum, EcoKitchen has proven capable of adding a significant contribution to the detection of the earliest impairments in HD, which in turn may facilitate an improvement in the management of these deficits – prolonging functioning at work, increasing social integration, and fostering independence in premanifest individuals (Reilmann et al., 2014). In our view, the several parameters computed from EcoKitchen and the way they relate to the different executive skills, to the self-reported functional incapacities and to the HD clinical symptomatology, can work as a proxy of the patients’ global cognitive and functional status and be extremely relevant to the design and implementation of pharmacological and non-pharmacological approaches to tackle specific domains. Improving the quality of life of HD affected individuals and settling a solid ground for effective interventions with disease-modifying goals is one of the main aims in the HD research field. The EcoKitchen task has the potential to be included in that effort. As some authors suggest (Nehl et al., 2004; Eddy and Rickards, 2015), acknowledging that the cognitive component of HD has an additional negative impact on functional capacity makes way for compensatory strategies to be implemented to offset some of the effects of cognitive decline on functional abilities. The EcoKitchen seems to be prone to be used as a rehabilitation tool or cognitive stimulation method, as it provides quantitative data that can track any subtle individual changes in the performance of simple and complex executive demanding tasks that simulate everyday-life routines. Finally, a better understanding of the dynamics between cognition and function in HD will improve the standards of care of HD affected individuals and guide the choice of outcome measures for future studies.

### Limitations

Functional capacity as tackled by performance-based measures is not fully synonymous of everyday functioning (Moore et al., 2007), as observed behavior during simulated tasks may differ greatly from what the individual does spontaneously in the environment (Williams et al., 2015). Virtual reality tools often cannot fully replicate the uncertainties of everyday life nor the compensatory aids/strategies that the individual uses to obtain a successful performance (McGuire, 2014). However, the use of virtual reality tasks in clinical research has several gains compared to real world settings, namely in terms of affordability, safety, efficiency, applicability to a wide range of conditions, and facility of data capturing and scoring, among others (Allain et al., 2014; Ruse et al., 2014; Parsons et al., 2015). Another possible limitation of EcoKitchen is that it might be somewhat difficult to isolate which specific executive functions are impaired and contribute to the performance deficits observed, as the three blocks involve complex tasks that rely on multiple cognitive skills. Yet, the several correlations computed between EcoKitchen and the other cognitive and functional measures, namely the conventional executive test battery, may help to clarify what is in fact being measured – EcoKitchen was proved to be mostly related to psychomotor/processing speed, planning, attention, set-shifting and cognitive flexibility tests.

On the other hand, IAFAI was only used as a self-report functional measure due to logistic constraints. Consequently, IAFAI results might not have fully captured the participants’ functional status, as lack of insight/awareness or anosognosia have been often described in HD and represent a challenge for the use of self-report assessment methods (Ho et al., 2006; Sitek et al., 2014). As Hoth et al. (2007) claim, family member/friend/caregiver ratings can potentially provide more reliable information about patients’ deficits than do patients’ reports. However, other authors like Giovannetti et al. (2008) state that reliance on caregiver questionnaires to assess everyday action is also prone to bias and offers only a very gross assessment of performance. We tried to provide some additional information about the subjects’ functional status using the UHDRS-TFC scores (which is a rating done by a neurologist). Furthermore, we were interested to see if there were disparities between the patients’ perception about their functional status and their objective performance in EcoKitchen – as it was the case for the HP group.

Finally, studies with larger sample sizes are needed to confirm the functionally significant executive deficits observed in HP individuals. Also, caution must be taken regarding the conclusions withdrawn from the comparisons of the two clinical groups, as differences in age and education level were detected between the HD and HP participants enrolled in our study and these variables might explain at least in part the differences observed in their performance. Moreover, future studies with other clinical conditions will further validate EcoKitchen as a sensitive assessment tool for patients with functional impairment due to executive deficits.

## Conclusion

We offer evidence that the EcoKitchen task is able to detect functionally significant deficits in early manifest non-demented HD patients and premanifest HD individuals. Given that this is an exploratory feasibility study, we would like to highlight several points that need to be addressed in future work to consubstantiate and expand the current findings. This new assessment tool must be validated in larger sample sizes, preferably including patients at different stages of HD severity, subdividing premanifest HD individuals into those far and close from estimated disease onset, and including other neurological conditions with similar brain driven cognitive impairments (e.g., Parkinson’s disease). Longitudinal studies would be beneficial to track individual and group changes along a timeline and to see whether targeting specific executive sub-domains with cognitive rehabilitation/enhancement strategies would have a positive impact in EcoKitchen performance. Moreover, as previously mentioned, performance-based measures are not fully synonymous of everyday functioning. Thus, a study exploring the relations between EcoKitchen performance and a real-life kitchen performance (preferably at home and not in a Lab setting) would help to corroborate its ecological validity.

From a clinical perspective, given the high variability demonstrated by the HD participants in the EcoKitchen task, individual assessments are considered necessary to have a nuanced characterization of each patient and accurately predict the person’s actual functional and executive status. This personalized and targeted approach is important to improve the efficacy of intervention and rehabilitation programs. In our view, the EcoKitchen can be an important asset contributing to a better understanding of the HD phenotype, clarifying the relation between cognition and daily living activities, facilitating the planning of tailored interventions, and, thus, improving the quality of life of those affected by HD.

## Data Availability

The datasets generated for this study are available on request to the corresponding author.

## Author Contributions

FJ wrote the manuscript. FJ and MP performed the statistical analysis. FJ, MvA, MRS, MC-B, and CJ contributed to the conception and design of the study. AM, FP, HG, and MS developed the EcoKitchen task. FJ and CJ performed the assessments and data acquisition. FJ, MR, MP, MvA, MRS, MC-B, and CJ contributed to the analysis and interpretation of data. MR, MP, MvA, MRS, MC-B, and CJ critically revised the manuscript.

## Conflict of Interest Statement

The authors declare that the research was conducted in the absence of any commercial or financial relationships that could be construed as a potential conflict of interest.

## References

[B1] Albani G. Raspelli S. Carelli L. Morganti F. Weiss P. L. Kizony R. (2010). Executive functions in a virtual world: a study in Parkinson’s disease. *Stud. Health Technol. Inform.* 154 92–96. 10.3233/978-1-60750-561-7-92 20543277

[B2] Allain P. Foloppe D. A. Besnard J. Yamaguchi T. Etcharry-Bouyx F. Le Gall D. (2014). Detecting everyday action deficits in alzheimer’s disease using a nonimmersive virtual reality kitchen. *J. Int. Neuropsychol. Soc.* 20 468–477. 10.1017/S1355617714000344 24785240

[B3] Alves L. Simões M. R. Martins C. (2012). The estimation of premorbid intelligence levels among portuguese speakers: the irregular word reading test (TeLPI). *Arch. Clin. Neuropsychol.* 27 58–68. 10.1093/arclin/acr103 22138319

[B4] Attree E. A. Brooks B. M. Rose F. D. Andrews T. K. Leadbetter A. G. Clifford B. R. (1996). “Memory processes and virtual environments: i can’t remember what was there, but i can remember how i got there. implications for people with disabilities,” in *Proceedings of the First European Conference on Disability, Virtual Reality, and Associated Technology*, ed. Sharkey P. M. (Reading: ECDVRAT & University of Reading), 117–123.

[B5] Aylward E. H. Sparks B. F. Field K. M. Yallapragada V. Shpritz B. D. Rosenblatt A. (2004). Onset and rate of striatal atrophy in preclinical huntington disease. *Neurology* 63 66–72. 10.1212/01.WNL.0000132965.14653.D1 15249612

[B6] Baake V. Reijntjes R. H. A. M. Dumas E. M. Thompson J. C. Registry Investigators of the European Huntington’s Disease Network and Roos R. A. C. (2017). Cognitive decline in huntington’s disease expansion gene carriers. *Cortex* 95 51–62. 10.1016/j.cortex.2017.07.017 28843844

[B7] Balci F. Day M. Rooney A. Brunner D. (2009). Disrupted temporal control in the R6/2 mouse model of huntington’s disease. *Behav. Neurosci.* 123 1353–1358. 10.1037/a0017650 20001119

[B8] Baum C. Edwards D. F. (1993). Cognitive performance in senile dementia of the alzheimer’s type: the kitchen task assessment. *Am. J. Occup. Ther.* 47 431–436. 10.5014/ajot.47.5.431 8498467

[B9] Beck A. T. Steer R. A. Brown G. K. (1996). *Manual for the Beck Depression Inventory-II.* San Antonio, TX: Psychological Corporation.

[B10] Beglinger L. J. O’Rourke J. J. F. Wang C. Langbehn D. R. Duff K. Paulsen J. S. (2010). Earliest functional declines in huntington disease. *Psychiatry Res.* 178 414–418. 10.1016/j.psychres.2010.04.030 20471695 PMC3629818

[B11] Bialystok E. Craik F. I. M. Stefurak T. (2008). Planning and task management in parkinson’s disease: differential emphasis in dual-task performance. *J. Int. Neuropsychol. Soc.* 14 257–265. 10.1017/S1355617708080296 18282323

[B12] Burgess P. W. Alderman N. Evans J. Emslie H. Wilson B. A. (1998). The ecological validity of tests of executive function. *J. Int. Neuropsychol. Soc.* 4 547–558. 10.1017/S1355617798466037 10050359

[B13] Burgess P. W. Alderman N. Forbes C. Costello A. Coates L. M.-A. Dawson D. R. (2006). The case for the development and use of “ecologically valid” measures of executive function in experimental and clinical neuropsychology. *J. Int. Neuropsychol. Soc.* 12 194–209. 10.1017/S1355617706060310 16573854

[B14] Campos R. C. Gonçalves B. (2011). The portuguese version of beck depression inventory-II (BDI-II): preliminary psychometric data with two non clinical samples. *Eur. J. Psychol. Assess.* 27 258–264. 10.1027/1015-5759/a000072

[B15] Cavaco S. Gonçalves A. Pinto C. Almeida E. Gomes F. Moreira I. (2013a). Semantic fluency and phonemic fluency: regression-based norms for the portuguese population. *Arch. Clin. Neuropsychol.* 28 262–271. 10.1093/arclin/act001 23341434

[B16] Cavaco S. Gonçalves A. Pinto C. Almeida E. Gomes F. Moreira I. (2013b). Trail making test: regression-based norms for the portuguese population. *Arch. Clin. Neuropsychol.* 28 189–198. 10.1093/arclin/acs115 23299183

[B17] Chaytor N. Schmitter-Edgecombe M. Burr R. (2006). Improving the ecological validity of executive functioning assessment. *Arch. Clin. Neuropsychol.* 21 217–227. 10.1016/j.acn.2005.12.002 16554143

[B18] Craik F. I. M. Bialystok E. (2006). Planning and task management in older adults: cooking breakfast. *Mem. Cogn.* 34 1236–1249. 10.3758/BF03193268 17225505

[B19] Downing N. R. Kim J.-I. Williams J. K. Long J. D. Mills J. A. Paulsen J. S. (2014). WHODAS 2.0 in prodromal huntington disease: measures of functioning in neuropsychiatric disease. *Eur. J. Hum. Genet.* 22 958–963. 10.1038/ejhg.2013.275 24327189 PMC4350592

[B20] Dumas E. M. van den Bogaard S. J. Middelkoop H. A. Roos R. A. (2013). A review of cognition in huntington’s disease. *Front. Biosci.* 5:1–18. 10.2741/S355 23277034

[B21] Eddy C. M. Rickards H. E. (2015). Cognitive deficits predict poorer functional capacity in huntington’s disease: but what is being measured? *Neuropsychology* 29 268–273. 10.1037/neu0000134 25110931

[B22] Folstein S. E. Abbott M. H. Franz M. L. Huang S. Chase G. A. Folstein M. F. (1984). Phenotypic heterogeneity in huntington disease. *J. Neurogenet.* 1 175–184. 10.3109/01677068409107083 6242167

[B23] Freitas S. Simões M. R. Alves L. Santana I. (2011). Montreal cognitive assessment (MoCA): normative study for the portuguese population. *J. Clin. Exp. Neuropsychol.* 33 989–996. 10.1080/13803395.2011.589374 22082082

[B24] Frisch S. Förstl S. Legler A. Schöpe S. Goebel H. (2012). The interleaving of actions in everyday life multitasking demands. *J. Neuropsychol.* 6 257–269. 10.1111/j.1748-6653.2012.02026.x 22329754

[B25] Giovannetti T. Bettcher B. M. Brennan L. Libon D. J. Kessler R. K. Duey K. (2008). Coffee with jelly or unbuttered toast: commissions and omissions are dissociable aspects of everyday action impairment in alzheimer’s disease. *Neuropsychology* 22 235–245. 10.1037/0894-4105.22.2.235 18331166

[B26] Godefroy O. Azouvi P. Robert P. Roussel M. LeGall D. Meulemans T. (2010). Dysexecutive syndrome: diagnostic criteria and validation study. *Ann. Neurol.* 68 855–864. 10.1002/ana.22117 21194155

[B27] Hamilton J. M. Salmon D. P. Corey-Bloom J. Gamst A. Paulsen J. S. Jerkins S. (2003). Behavioural abnormalities contribute to functional decline in huntington’s disease. *J. Neurol. Neurosurg. Psychiatry* 74 120–122. 10.1136/jnnp.74.1.120 12486282 PMC1738208

[B28] Heaton R. K. (1981). *A Manual for the Wisconsin Card Sorting Test.* Odessa, FL: Psychological Assessment Resources.

[B29] Heitz R. P. (2014). The speed-accuracy tradeoff: history, physiology, methodology, and behavior. *Front. Neurosci.* 8:150. 10.3389/fnins.2014.00150 24966810 PMC4052662

[B30] Ho A. Robbins A. O. G. Barker R. A. (2006). Huntington’s disease patients have selective problems with insight. *Mov. Disord.* 21 385–389. 10.1002/mds.20739 16211608

[B31] Hoth K. F. Paulsen J. S. Moser D. J. Tranel D. Clark L. A. Bechara A. (2007). Patients with huntington’s disease have impaired awareness of cognitive, emotional, and functional abilities. *J. Clin. Exp. Neuropsychol.* 29 365–376. 10.1080/13803390600718958 17497560

[B32] Huntington Study Group (1996). Unified huntington’s disease rating scale: reliability and consistency. *Mov. Disord.* 11 136–142. 10.1002/mds.870110204 8684382

[B33] Kawano N. Iwamoto K. Ebe K. Aleksic B. Noda A. Umegaki H. (2012). Slower adaptation to driving simulator and simulator sickness in older adults aging clinical and experimental research. *Aging Clin. Exp. Res.* 24 285–289. 10.1007/BF03325260 23114558

[B34] Keefe R. S. E. Davis V. G. Atkins A. S. Vaughan A. Patterson T. Narasimhan M. (2016). Validation of a computerized test of functional capacity. *Schizophr. Res.* 175 90–96. 10.1016/j.schres.2016.03.038 27091656 PMC4958510

[B35] Langbehn D. R. Brinkman R. R. Falush D. Paulsen J. S. Hayden M. R. An International Huntington’s Disease Collaborative Group (2004). A new model for prediction of the age of onset and penetrance for huntington’s disease based on CAG length. *Clin. Genet.* 65 267–277. 10.1111/j.1399-0004.2004.00241.x 15025718

[B36] Lemiere J. Decruyenaere M. Evers-Kiebooms G. Vandenbussche E. Dom R. (2002). Longitudinal study evaluating neuropsychological changes in so-called asymptomatic carriers of the huntington’s disease mutation after 1 year. *Acta Neurol. Scand.* 106 131–141. 10.1034/j.1600-0404.2002.01192.x 12174172

[B37] Lezak M. D. (1982). The problem of assessing executive functions. *Int. J. Psychol.* 17 281–297. 10.1080/00207598208247445

[B38] Lezak M. D. Howieson D. B. Bigler E. D. Tranel D. (2012). *Neuropsychological Assessment*, 5th Edn New York, NY: Oxford University Press.

[B39] McCusker E. Loy C. T. (2014). The many facets of unawareness in huntington disease. *Tremor Other Hyperkinet. Mov.* 4:257. 10.7916/D8FJ2FD3 25411649 PMC4231168

[B40] McGuire B. E. (2014). Assessing complex executive functions with computerized tests: is that toast burning? *Front. Behav. Neurosci.* 8:362. 10.3389/fnbeh.2014.00362 25374518 PMC4205925

[B41] Mehrabi N. F. Waldvogel H. J. Tippett L. J. Hogg V. M. Synek B. J. Faull R. L. M. (2016). Symptom heterogeneity in huntington’s disease correlates with neuronal degeneration in the cerebral cortex. *Neurobiol. Dis.* 96 67–74. 10.1016/j.nbd.2016.08.015 27569581

[B42] Moore D. J. Palmer B. W. Patterson T. L. Jeste D. V. (2007). A review of performance-based measures of functional living skills. *J. Psychiatr. Res.* 41 97–118. 10.1016/j.jpsychires.2005.10.008 16360706

[B43] Mörkl S. Müller N. J. Blesl C. Wilkinson L. Tmava A. Wurm W. (2016). Problem solving, impulse control and planning in patients with early- and late-stage huntington’s disease. *Eur. Arch. Psychiatry Clin. Neurosci.* 266 663–671. 10.1007/s00406-016-0707-4 27372072 PMC5037143

[B44] Nasreddine Z. S. Phillips N. A. Bédirian V. Charbonneau S. Whitehead V. Collin I. (2005). The montreal cognitive assessment, MoCA: a brief screening tool for mild cognitive impairment. *J. Am. Geriatr. Soc.* 53 695–699. 10.1111/j.1532-5415.2005.53221.x 15817019

[B45] Nehl C. Paulsen J. S. The Huntington Study Group (2004). Cognitive and psychiatric aspects of huntington disease contribute to functional capacity. *J. Nerv. Ment. Dis.* 192 72–74. 10.1097/01.nmd.0000106004.67587.57 14718780

[B46] Nicoll D. R. Pirogovsky E. Woods S. P. Holden H. M. Filoteo J. V. Gluhm S. (2014). “Forgetting to remember” in huntington’s disease: a study of laboratory, semi-naturalistic, and self-perceptions of prospective memory. *J. Int. Neuropsychol. Soc.* 20 192–199. 10.1017/S1355617713001355 24351166

[B47] Nimmagadda S. R. Agrawal N. Worrall-Davies A. Markova I. Rickards H. (2011). Determinants of irritability in huntington’s disease. *Acta Neuropsychiatr.* 23 309–314. 10.1111/j.1601-5215.2011.00563.x 25380043

[B48] Norris G. Tate R. L. (2000). The behavioural assessment of the dysexecutive syndrome (BADS): ecological, concurrent and construct validity. *Neuropsychol. Rehabil.* 10 33–45. 10.1080/096020100389282

[B49] Novak M. J. U. Tabrizi S. J. (2010). Huntington’s disease. *BMJ* 340:c3109. 10.1136/bmj.c3109 20591965

[B50] Novak M. J. U. Tabrizi S. J. (2011). Huntington’s disease: clinical presentation and treatment. *Int. Rev. Neurobiol.* 98 297–323. 10.1016/B978-0-12-381328-2.00013-4 21907093

[B51] O’Keeffe G. C. Michell A. W. Barker R. A. (2009). Biomarkers in huntington’s and parkinson’s disease. *Ann. N. Y. Acad. Sci.* 1180 97–110. 10.1111/j.1749-6632.2009.04943.x 19906264

[B52] Oldfield R. C. (1971). The assessment and analysis of handedness: the edinburgh inventory. *Neuropsychologia* 9 97–113. 10.1016/0028-3932(71)90067-4 5146491

[B53] Parsons T. D. Carlew A. R. Magtoto J. Stonecipher K. (2015). The potential of function-led virtual environments for ecologically valid measures of executive function in experimental and clinical neuropsychology. *Neuropsychol. Rehabil.* 27 777–807. 10.1080/09602011.2015.1109524 26558491

[B54] Paulsen J. S. (2010). Early detection of huntington disease. *Fut. Neurol.* 5 85–104. 10.2217/fnl.09.78 24348095 PMC3860286

[B55] Paulsen J. S. (2011). Cognitive impairment in huntington disease: diagnosis and treatment. *Curr. Neurol. Neurosci. Rep.* 11 474–483. 10.1007/s11910-011-0215-x 21861097 PMC3628771

[B56] Paulsen J. S. Hayden M. Stout J. C. Langbehn D. R. Aylward E. Ross C. A. (2006). Preparing for preventive clinical trials: the predict-HD study. *Arch. Neurol.* 63 883–890. 10.1001/archneur.63.6.883 16769871

[B57] Poliakoff E. Smith-Spark J. H. (2008). Everyday cognitive failures and memory problems in parkinson’s patients without dementia. *Brain Cogn.* 67 340–350. 10.1016/j.bandc.2008.02.004 18358582

[B58] Rao J. A. Harrington D. L. Durgerian S. Reece C. Mourany L. Koenig K. (2014). Disruption of response inhibition circuits in prodromal huntington disease. *Cortex* 58 72–85. 10.1016/j.cortex.2014.04.018 24959703 PMC4227536

[B59] Reilmann R. Leavitt B. R. Ross C. A. (2014). Diagnostic criteria for huntington’s disease based on natural history. *Mov. Disord.* 29 1335–1341. 10.1002/mds.26011 25164527

[B60] Roos R. A. (2010). Huntington’s disease: a clinical review. *Orphanet J. Rare Dis.* 5:40. 10.1186/1750-1172-5-40 21171977 PMC3022767

[B61] Rosas H. D. Hevelone N. D. Zaleta A. K. Greve D. N. Salat D. H. Fischl B. (2005). Regional cortical thinning in preclinical huntington disease and its relationship to cognition. *Neurology* 65 745–747. 10.1212/01.wnl.0000174432.87383.87 16157910

[B62] Rosenblatt A. (2007). Neuropsychiatry of huntington’s disease. *Dialogues Clin. Neurosci.* 9 191–197. 17726917 10.31887/DCNS.2007.9.2/arosenblatt PMC3181855

[B63] Ross C. A. Aylward E. H. Wild E. J. Langbehn D. R. Long J. D. Warner J. H. (2014a). Huntington disease: natural history, biomarkers and prospects for therapeutics. *Nat. Rev. Neurol.* 10 204–216. 10.1038/nrneurol.2014.24 24614516

[B64] Ross C. A. Pantelyat A. Kogan J. Brandt J. (2014b). Determinants of functional disability in huntington’s disease: role of cognitive and motor dysfunction. *Mov. Disord.* 29 1351–1358. 10.1002/mds.26012 25216368 PMC4197404

[B65] Rothlind J. C. Bylsma F. W. Peyser C. Folstein S. E. Brandt J. (1993). Cognitive and motor correlates of everyday functioning in early huntington’s disease. *J. Nerv. Ment. Dis.* 181 194–199. 10.1097/00005053-199303000-00008 8445379

[B66] Royall D. R. Lauterbach E. C. Kaufer D. Malloy P. Coburn K. L. Black K. J. (2007). The cognitive correlates of functional status: a review from the committee on research of the american neuropsychiatric association. *J. Neuropsychiatry Clin. Neurosci.* 19 249–265. 10.1176/appi.neuropsych.19.3.249 17827410

[B67] Ruse S. A. Davis V. G. Atkins A. S. Krishnan K. R. R. Fox K. H. Harvey P. D. (2014). Development of a virtual reality assessment of everyday living skills. *J. Vis. Exp.* 86:e51405. 10.3791/51405 24798174 PMC4174921

[B68] Sheppard D. P. Pirogovsky-Turk E. Woods S. P. Holden H. M. Nicoll D. R. Filoteo J. V. (2017). Everyday functioning in huntington’s disease?: a laboratory-based study of financial management capacity. *Appl. Neuropsychol.* 24 176–182. 10.1080/23279095.2015.1125904 27077945

[B69] Shoulson I. Fahn S. (1979). Huntington disease: clinical care and evaluation. *Neurology* 29 1–3. 10.1212/WNL.29.1.1 154626

[B70] Shoulson I. Young A. B. (2011). Milestones in huntington disease. *Mov. Disord.* 26 1127–1133. 10.1002/mds.23685 21626556

[B71] Sitek E. J. Sołtan W. Wieczorek D. Schinwelski M. Robowski P. Reilmann R. (2011). Self-awareness of motor dysfunction in patients with huntington’s disease in comparison to parkinson’s disease and cervical dystonia. *J. Int. Neuropsychol. Soc.* 17 788–795. 10.1017/S1355617711000725 21729402

[B72] Sitek E. J. Thompson J. C. Craufurd D. Snowden J. S. (2014). Unawareness of deficits in huntington’s disease. *J. Huntingtons Dis.* 3 125–135. 10.3233/JHD-140109 25062855

[B73] Smith A. (1982). *Symbol Digits Modalities Test (SDMT) Manual.* Los Angeles, CA: Western Psychological Services.

[B74] Snowden J. S. Craufurd D. Griffiths H. L. Neary D. (1998). Awareness of involuntary movements in huntington disease. *Arch. Neurol.* 55 801–805. 10.1001/archneur.55.6.801 9626771

[B75] Sohlberg M. M. Mateer C. A. (2001). *Cognitive Rehabilitation: An Integrative Neuropsychological Approach.* New York, NY: Guilford Press.

[B76] Sousa L. B. Prieto G. Vilar M. Firmino H. Simões M. R. (2015). The adults and older adults functional assessment inventory: a rasch model analysis. *Res. Aging* 37 787–814. 10.1177/0164027514564469 25651593

[B77] Stout J. C. Glikmann-Johnston Y. Andrews S. C. (2016). Cognitive assessment strategies in huntington’s disease research. *J. Neurosci. Methods* 265 19–24. 10.1016/j.jneumeth.2015.12.007 26719240

[B78] Stout J. C. Paulsen J. S. Queller S. Solomon A. C. Whitlock K. B. Campbell J. C. (2011). Neurocognitive signs in prodromal huntington disease. *Neuropsychology* 25 1–14. 10.1037/a0020937 20919768 PMC3017660

[B79] Stout J. C. Queller S. Baker K. N. Cowlishaw S. Sampaio C. Fitzer-Attas C. (2014). HD-CAB: a cognitive assessment battery for clinical trials in huntington’s disease. *Mov. Disord.* 29 1281–1288. 10.1002/mds.25964 25209258

[B80] Stout J. C. Weaver M. Solomon A. C. Queller S. Hui S. Johnson S. A. (2007). Are cognitive changes progressive in prediagnostic HD? *Cogn. Behav. Neurol.* 20 212–218. 10.1097/WNN.0b013e31815cfef8 18091069

[B81] Stroop J. R. (1935). Studies of interference in serial verbal reactions. *J. Exp. Psychol.* 18 643–662. 10.1037/0096-3445.121.1.15

[B82] Tabrizi S. J. Langbehn D. R. Leavitt B. R. Roos R. A. Durr A. Craufurd D. (2009). Biological and clinical manifestations of huntington’s disease in the longitudinal TRACK-HD study: cross-sectional analysis of baseline data. *Lancet Neurol.* 8 791–801. 10.1016/S1474-4422(09)70170-X 19646924 PMC3725974

[B83] Tabrizi S. J. Scahill R. I. Durr A. Roos R. A. C. Leavitt B. R. Jones R. (2011). Biological and clinical changes in premanifest and early stage huntington’s disease in the TRACK-HD study: the 12-month longitudinal analysis. *Lancet Neurol.* 10 31–42. 10.1016/S1474-4422(10)70276-3 21130037

[B84] Tabrizi S. J. Scahill R. I. Owen G. Durr A. Leavitt B. R. Roos R. A. (2013). Predictors of phenotypic progression and disease onset in premanifest and early-stage huntington’s disease in the TRACK-HD study: analysis of 36-month observational data. *Lancet Neurol.* 12 637–649. 10.1016/S1474-4422(13)70088-7 23664844

[B85] Tanguay A. N. Davidson P. S. R. Guerrero Nuñez K. V. Ferland M. B. (2014). Cooking breakfast after a brain injury. *Front. Behav. Neurosci.* 8:272. 10.3389/fnbeh.2014.00272 25228863 PMC4151095

[B86] van Asselen M. Júlio F. Januário C. Bobrowicz-Campos E. Almeida I. Cavaco S. (2012). Scanning patterns of faces do not explain impaired emotion recognition in huntington disease: evidence for a high-level mechanism. *Front. Psychol.* 3:31. 10.3389/fpsyg.2012.00031 22355293 PMC3280621

[B87] Waldvogel H. J. Kim E. H. Thu D. C. Tippett L. J. Faull R. L. (2012). New perspectives on the neuropathology in huntington’s disease in the human brain and its relation to symptom variation. *J. Huntingtons Dis.* 1 143–153. 10.3233/JHD-2012-120018 25063328

[B88] Wechsler D. (1997). *WAIS-III Administration and Scoring Manual*, 3rd Edn San Antonio, TX: The Psychological Corporation.

[B89] Wechsler D. (2008). *Escala de Inteligência de Wechsler Para Adultos - Manual (3a edição) [Wechsler Adult Intelligence Scale -]*, 3rd Edn Lisboa: Cegoc.

[B90] Weir D. W. Sturrock A. Leavitt B. R. (2011). Development of biomarkers for huntington’s disease. *Lancet Neurol.* 10 573–590. 10.1016/S1474-4422(11)70070-9 21601164

[B91] Williams J. Downing N. Vaccarino A. L. Guttman M. Paulsen J. S. (2011). Self-reports of day-to-day function in a small cohort of people with prodromal and early HD. *PLoS Curr.* 3:RRN1254. 10.1371/currents.RRN1254 21901173 PMC3154838

[B92] Williams J. K. Kim J. Downing N. Farias S. Harrington D. L. Long J. D. (2015). Everyday cognition in prodromal huntington disease. *Neuropsychology* 29 255–267. 10.1037/neu0000102 25000321 PMC4286521

[B93] Wilson B. A. Alderman N. Burgess P. W. Emslie H. Evans J. J. (1996). *Behavioural Assessment of Dysexecutive Syndrome.* London: Thames Valley Test Company.

[B94] Wilson B. A. Evans J. J. Emslie H. Alderman N. Burgess P. (1998). The development of an ecologically valid test for assessing patients with a dysexecutive syndrome. *Neuropsychol. Rehabil.* 8 213–228. 10.1080/713755570

[B95] Zhang L. Abreu B. C. Seale G. S. Masel B. Christiansen C. H. Ottenbacher K. J. (2003). A virtual reality environment for evaluation of a daily living skill in brain injury rehabilitation: reliability and validity. *Arch. Phys. Med. Rehabil.* 84 1118–1124. 10.1016/S0003-9993(03)00203-X 12917848

